# Predictors of recurrence after catheter ablation and electrical cardioversion of atrial fibrillation: an umbrella review of meta-analyses

**DOI:** 10.1093/europace/euac143

**Published:** 2022-08-29

**Authors:** Emmanouil Charitakis, Elena Dragioti, Maria Stratinaki, Dafni Korela, Stylianos Tzeis, Henrik Almroth, Ioan Liuba, Anders Hassel Jönsson, Georgios Charalambous, Lars O Karlsson, Dimitrios Tsartsalis

**Affiliations:** Department of Cardiology and Department of Health, Medicine and Caring Sciences, Linköping University, Linköping 58185, Sweden; Pain and Rehabilitation Centre and Department of Health, Medicine and Caring Sciences, Linköping University, Linköping 58758, Sweden; Department of Cardiology, Venizeleio General Hospital, Heraklion, Crete 71409, Greece; Department of Cardiology, Venizeleio General Hospital, Heraklion, Crete 71409, Greece; Mitera Hospital, Hygeia Group, Athens 15123, Greece; Department of Cardiology and Department of Health, Medicine and Caring Sciences, Linköping University, Linköping 58185, Sweden; Department of Cardiology and Department of Health, Medicine and Caring Sciences, Linköping University, Linköping 58185, Sweden; Department of Cardiology and Department of Health, Medicine and Caring Sciences, Linköping University, Linköping 58185, Sweden; Department of Emergency Medicine, ‘Hippokration’ Hospital, Athens, Vasilissis Sofias 114, 11527 Athens, Greece; Department of Cardiology and Department of Health, Medicine and Caring Sciences, Linköping University, Linköping 58185, Sweden; Department of Emergency Medicine, ‘Hippokration’ Hospital, Athens, Vasilissis Sofias 114, 11527 Athens, Greece

**Keywords:** Catheter ablation • Atrial fibrillation, Umbrella review, Electrical cardioversion

## Abstract

**Aims:**

The recurrence rates after catheter ablation (CA) and direct current (DC) cardioversion remain high, although they have been established treatments of rhythm control of atrial fibrillation (AF). This umbrella review systematically appraises published meta-analyses of both observational and randomized controlled trials (RCTs) for the association of risk and protective factors for arrhythmia recurrence after CA and DC cardioversion of AF.

**Methods and results:**

Three bibliographic databases were searched up to June 2021. Evidence of association was rated as convincing, highly suggestive, suggestive, weak, or not significant with respect to observational studies and as high, moderate, low, or very low with respect to RCTs, according to established criteria. Thirty-one meta-analyses were included. Of the 28 associations between CA and the risk of arrhythmia recurrence, none presented convincing evidence, and only the time from diagnosis to ablation over 1 year provided highly suggestive evidence. The association between hypertension and metabolic profile provided suggestive evidence. The associations of Class IC and III antiarrhythmic drugs use with the recurrence after DC cardioversion were supported by an intermediate level of evidence.

**Conclusion:**

Although AF is a major health issue, few risk- and protective factors for AF recurrence have been identified. None of these factors examined were supported by convincing evidence, whereas established factors such as female gender and left atrial volume showed only weak association. An early CA strategy combined with treatment of metabolic syndrome and hypertension prior to CA may reduce the risk of arrhythmia recurrence. The use of antiarrhythmics can increase the success rate of DC cardioversion.

**Systematic review registration:**

PROSPERO registry number: CRD42021270613.

**What’s new?**

- Our study is the first umbrella review to systematically appraise published meta-analyses of both observational and randomized controlled trials for the association of risk and protective factors for arrhythmia recurrence after catheter ablation (CA) and direct current (DC) cardioversion of atrial fibrillation (AF).
- Few risk- and protective factors of arrhythmia recurrence after CA and DC cardioversion of AF have been identified.
- None of the factors examined showed association with AF recurrence supported by convincing evidence.
- An early CA strategy and management of metabolic syndrome and hypertension prior to CA can reduce the risk of arrhythmia recurrence.
- The use of antiarrhythmic drugs, mainly amiodarone, flecainide, and propafenone, can increase the success rate of DC cardioversion.

## Introduction

The prevalence of atrial fibrillation (AF) among adults aged older than 55 years of age in the European Union was estimated at ∼8.8 million in 2010. An increase to around 14 million is forecast by 2060.^1^ These numbers are influenced by both unmodifiable factors such as advancing age and modifiable risk factors associated with modern lifestyle such as high body mass index (BMI), physical inactivity, hypertension, obstructive sleep apnoea syndrome (OSAS) and psychosocial stress.^2^ Atrial fibrillation is associated with an increased risk of death, primarily from heart failure (HF) and stroke.^3^

Currently, stroke prevention with oral anticoagulation has reduced the risk of death in patients with AF. However, many patients remain symptomatic as well the risk of HF.^4^

Synchronized direct current (DC) cardioversion is an integral part of rhythm control in patients with AF.^5^ Direct cardioversion terminates AF in over 90% of cases with rare complications.^6^ Nevertheless, the recurrence rate of AF is high, approximately 50% in the first two weeks and 65% in the first year after cardioversion.^7^ Consequently, various alternatives such as the ‘wait and see’ approach in patients with recent-onset AF and the use of hybrid treatment, combining DC cardioversion with pre-treatment with antiarrhythmic drugs (AADs) have been proposed.^5,7^ Identifying predictors of successful DC cardioversion is crucial. So far, age, functional class of the patients, and eventual prior treatment with AAD have been identified as predictors of successful cardioversion.^6^

Catheter ablation (CA) is an established and safe treatment option for rhythm control in patients with symptomatic AF.^3,8^ Pulmonary vein isolation (PVI) has been the basis of CA procedures for AF since the pioneering observation of induction of AF by ectopic beats from the pulmonary veins.^9,10^ CA has evolved substantially over the past few decades, with several different tools becoming accessible and different approaches being adopted. To date, none of the novel approaches have been proven superior to PVI alone.^11^ Success rates vary between 50 and 80%, depending on the type of AF (paroxysmal or persistent).^11,12^ The likelihood of successful CA is related to both procedure and patient characteristics.^11,13^ Given the moderate success rate of CA in patients with AF, several studies have explored different factors such as gender, AF duration, age, as predictors of recurrence post-CA of AF.^14–16^ No study has yet collectively summarized, evaluated, and graded the evidence of the clinically applicable research on this topic.

The aim of this paper is to summarize the existing evidence on risk and protective factors associated with the risk of recurrence after CA and DC cardioversion of AF across published meta-analyses through an umbrella review. Following best research practices, the evidence from the available meta-analyses on this topic was ranked based on sample size, the strength of the association, and the presence of various biases.^17,18^

## Methods

The Preferred Reporting Items for Systematic Reviews and Meta-Analyses (PRISMA)^19^ reporting guidelines and the Meta-Analysis of Observational Studies in Epidemiology (MOOSE) guidelines^20^ (see Supplementary material online, *Appendix S1* in the Supplement) were used in this study. The study protocol was registered in the PROSPERO database. (CRD42021270613).

### Data selection, search strategy, and selection criteria

Bibliographic databases (PubMed, Web of Science, and the Cochrane Database of Systematic Reviews) were searched up to May 28, 2021, to identify systematic reviews with meta-analysis of observational or randomized controlled trials (RCTs) that examined associations between non-genetic risk or protective factors, with the risk of recurrence after CA or/and DC cardioversion of AF as a primary or secondary endpoint. Our search strategy was broad to identify all eligible studies using terms related to AF and meta-analysis (see Supplementary material online, *Appendix S2* in the Supplement). The reference lists of eligible studies were also examined to identify additional studies.

Two researchers (D.K., M.S.) independently conducted the initial search for relevant articles. The full texts of the retrieved articles were further examined for eligibility by the same researchers. Records of any discrepancies regarding the eligibility for inclusion were reviewed by a third investigator (E.C.), and disagreements were resolved through discussion.

We included only peer-reviewed meta-analyses of RCTs or observational studies with either a cohort, case-control, or nested case-control study design and examined any association between recurrence of AF after CA or DC cardioversion and risk- or protective factors. In the case of multiple meta-analyses evaluating the same risk and/or protective factors, we kept the meta-analysis with the largest number of included studies.^21^ All reported outcomes for eligible meta-analyses were considered for inclusion.

Meta-analyses were excluded for (i) study designs other than those mentioned above (cross-sectional, letter to the editor); (ii) a non-systematic selection of observational studies or RCTs and non-systematic reviews; (iii) examining genetic variants as risk factors for recurrence after CA or DC cardioversion; (iv) studies published in languages other than English; (v) insufficient data for quantitative synthesis were provided; or (vi) they presented study-specific effects estimates other than odds ratio (OR), hazard ratio (HR), or relative risk (RR), i.e. mean difference. Reasons for exclusion after full-text assessment were listed in the supplementary material (see Supplementary material online, *Appendix S3* in the Supplement).

### Data extraction

Two researchers (D.K., M.S.) independently performed data extraction from each eligible article using a predefined extraction form (EXCEL 365). Any disagreements were resolved through discussion. For each meta-analysis, the collected variables included: first authors’ name, year of publication, journal, standard identifier (DOI), number of primary studies, total sample size, and risk or protective factors assessed. For each primary study, we collected: the first authors´ name, year of publication, study design, sample size (exposure and non-exposure), and relative risk estimates (i.e. HR, OR, RR) with the subsequent 95% confidence interval (CI).

### Quality assessment

Risk of bias (RoB) was assessed using the AMSTAR2 tool (A Measurement Tool to Assess systematic Reviews, available at https://amstar.ca/Amstar-2.php). This tool was designed to assess meta-analyses of both randomized and non-randomized studies and applies criteria within ten original domains. The RoB assessment was performed by two reviewers (D.T., M.S.) and checked by two others (E.C., D.T.).^22^

### Data synthesis and analysis

The effect size (ES) of different studies reported in each meta-analysis was extracted for each association. We re-measured the pooled effect sizes and 95% CIs employing random-effects models^23^ and examined inter-study heterogeneity using the I^2^ statistic.^24^ The small-study effect bias was evaluated with the Egger regression asymmetry test and random-effects summary effect size to determine whether smaller studies produced larger effect sizes compared to larger studies.^25,26^ Finally, the excess significance bias was measured to decide whether the observed number of studies with statistically significant results differed from the anticipated number of studies with statistically significant results.^27^ The anticipated number of statistically significant studies per association was calculated by summing the statistical power estimates for each component study. The power estimates of each component study depend on the reasonable ES for the examined association, which are assumed to be the ES of the largest study (i.e. the smallest standard error) per association. A *P*-value ≤ 0.10 was considered statistically significant for excess significance bias.^27^ All analyses were performed using Stata 17.0 (StataCorp, College Station, TX) and R v.4.0.3 (The R Foundation for Statistical Computing, Auckland, NZ).

Suitable associations from observational studies were categorized into five classes, according to the power of the evidence of possible risk or protective factors: convincing (Class I), highly suggestive (Class II), suggestive (Class III), weak (Class IV), and not significant (NS) (eTable 1, Supplementary material online, *Appendix S4* in the Supplement), consistent with previous umbrella reviews.^28^

Regarding RCTs, the credibility of evidence was categorized according to the summary effect (*P*-value <0.01, 0.01 ≤ *P*-value < 0.05, *P*-value ≥ 0.05), 95% prediction interval (excluding the null or not), presence of large heterogeneity (I² >50%), small study effects (*P* < 0.10), and excess significance (*P* < 0.10).^29^ Additionally, GRADE (Grading of Recommendations Assessment, Development, and Evaluation) levels of evidence (GLE) using a modified concrete set of rules was also employed as previously described.^30,31^ Four areas were evaluated: (i) imprecision, by the number of participants in the pooled analysis (if 100–199 participants, GLE was downgraded by 1 level; if <100 participants, downgraded by two levels); (ii) RoB original study quality, by the proportion of RCTs included in the pooled analysis with low RoB for randomization and observer blinding (if ≥ 75% of RCTs had low RoB or RoB not reported, GLE was downgraded by 1 level); (iii) inconsistency, by heterogeneity (if I² >75%, downgraded by 1 level); and (iv) RoB review quality, by the responses to AMSTAR2 questionnaire (if moderate quality, downgraded by 1 level; if low or critically low quality, downgraded by two levels). Then, reviews were classified as high, moderate, low, or very low according to GLE (eTable 2, Supplementary material online, *Appendix S4* in the Supplement).

## Results

### Literature search

**Figure 1:**
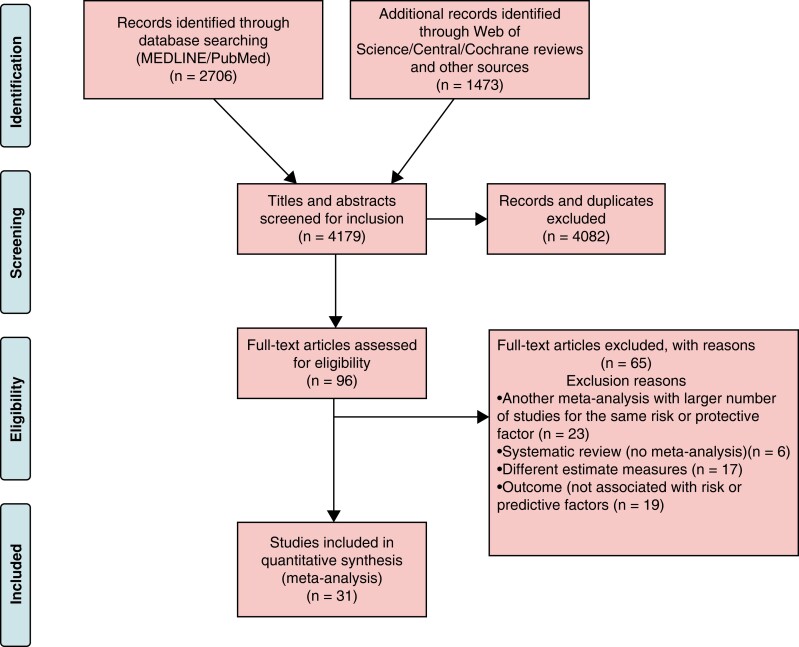
Flowchart of the study selection process.

The initial search yielded 4179 publications. After title and abstract assessment, 96 eligible articles were identified. Then 65 articles were excluded after a full-text review (see Supplementary material online, *Appendix S3*), and 31 articles were included for analysis. Twenty-six studies evaluated risk and protective factors for recurrence of AF post-CA and reported 28 associations, while six studies assessed risk- and protective factors for AF recurrence after DC cardioversion and reported 25 associations (*Figure 1*). Background factors of the included studies are presented in Supplementary material online, *Appendix S5* in the supplement.

### Risk and protective factors of recurrence post-catheter ablation of atrial fibrillation

The quality score of the meta-analyses of observational studies on risk and protective factors for recurrence of AF post-CA, following AMSTAR2, was low and critically low in 14 studies, moderate in six and high in six (*Table 1* and Supplementary material online, *Appendix S5* in the Supplement). The median number of studies included in the original meta-analyses was six (IQR = 5–10), the median number of patients was 1304 (IQR = 639–3806), and the median number of incidences of recurrence was 454 (IQR = 291–932).

**Table 1:** Predictors for AF recurrence, in meta-analyses of observational studies

Author, year	Predictor	Exposed/unexposed as in included MA	K	n/*n*	Metric	ES (95% CI)	*P*	PI include null value	I^2^	SSE	ESB	LS sign	CE	CES2 (*n* > 1000)	AMSTAR 2 quality
**Post-ablation**															
Chew, 2020	Diagnosis-to-Ablation Time (DAT)	DAT ≤ 1 year vs. DAT > 1 year	6	2220/4950	RR	0.729 (0.646, 0.822)	2.7 × 10^–7^	Yes	53.73%	No	NP	Yes	II	II	High
Lin 2013	Metabolic syndrome	Metabolic syndrome vs. no metabolic syndrome	5	1184/3320	RR	1.63 (1.25, 2.12)	2.9 × 10^–4^	Yes	54.3%	Yes	Yes	Yes	III	III	Low
Lin 2013	Hypertension	Hypertension vs. no hypertension	17	3249/11 430	RR	1.31 (1.13, 1.51)	2.6 × 10^–4^	Yes	47.5%	No	Yes	No	III	III	Low
Liu 2020	Burst-pacing post-CA	AF inducibility vs. AF non-inducibility	14	702/2628	RR	0.661 (0.585, 0.748)	4.3 × 10^–11^	Yes	0%	No	Yes	Yes	IV	II	Critically low
Zhuo 2020	Depression	depression vs. no depression	7	306/985	RR	2.245 (1.750, 2.879)	1.8 × 10^–20^	No	29.17%	Yes	Yes	Yes	IV	II	Critically low
Shukla, 2015	OSAS + CPAP	CPAP vs. no CPAP	7	494/1087	RR	0.585 (0.511 0.670)	1.7 × 10^–14^	Yes	0%	No	No	Yes	IV	II	Moderate
Li, 2014	OSAS	OSA vs. non-OSA	5	931/3743	RR	1.376 (1.205, 1.572)	2.6 × 10^–6^	No	10.22%	No	Yes	Yes	IV	III	High
Atti, 2019	Renal sympathetic denervation	Renal sympathetic denervation performed vs. not performed	6	222/432	RR	0.586 (0.472, 0.729)	1.4 × 10^–6^	Yes	0%	No	No	Yes	IV	III	Moderate
Zhuang, 2013	High BMI (≥25 kg/m2)	high BMI vs. normal BMI	8	804/2441	OR	1.558 (1.222, 1.987)	4.0 × 10^–4^	Yes	29.37%	No	NP	Yes	IV	III	Moderate
Correia 2019	LA stiffness	increased stiffness vs. decreased stiffness	3	340/649	HR	3.551 (1.805, 6.985)	2.0 × 10^–4^	Yes	66.84%	No	No	Yes	IV	III	Critically low
Pranata, 2019	*P*-wave duration	prolonged *P* wave duration vs. normal duration	6	278/787	OR	4.169 (2.101, 8.274)	4.5 × 10^–5^	Yes	71.38%	Yes	Yes	No	IV	III	High
Chen 2020	High-power short duration radiofrequency ablation	high-power short-duration vs. low-power long-duration	3	454/607	RR	1.184 (1.071, 1.308)	0.001	Yes	28.89%	No	Yes	Yes	IV	IV	High
Vallakati, 2017	Female sex	women vs. men	20	2755/9968	OR	1.198 (1.043, 1.377])	0.010	Yes	25.56%	No	NP	Yes	IV	IV	Moderate
Ng, 2011	OSAS	OSA vs. non-OSA	6	1157/3995	RR	1.251 (1.082, 1.447)	0.003	Yes	48.75%	No	No	Yes	IV	IV	Critically low
Lee, 2021	CKD	CKD vs. non-CKD	8	3267/23 566	OR	2.991 (1.518, 5.892)	0.002	Yes	87.95%	Yes	Yes	No	IV	IV	Critically low
Njoku 2018	LAV	larger LAVI vs. smaller LAVI	13	84/2693	OR	1.032 (1.012, 1.052)	0.002	Yes	80.16%	Yes	Yes	Yes	IV	IV	Critically low
Tse, 2018	Inter-atrial block	Inter-atrial block vs. no inter-atrial block	3	182/265	HR	2.586 (1.350, 4.955)	0.004	Yes	67.26%	No	No	Yes	IV	IV	Critically low
Peng 2020	RAAS	Use or not	15	NR/4302	RR	0.83 (0.7, 0.98)	0.026	Yes	68.5%	No	NA	No	IV	IV	Low
Lei 2018	Steroids	Use or not	5	NR/771	RR	0.67 (0.47, 0.95)	0.023	Yes	0%	No	NA	No	IV	IV	Critically low
Zhang, 2018	Prolonged corrected QT interval	prolonged corrected QT interval vs. control	3	366/415	HR	1.033 (0.987, 1.082)	0.162	Yes	86.77%	Yes	Yes	Yes	NS	NA	High
McLellan, 2013	Adenosine	acute reconnection vs. no reconnection	6	366/554	RR	0.913 (0.807,1.033)	0.148	Yes	0%	No	No	No	NS	NA	Critically low
Huang 2020	Low fluoroscopy	Low-fluoscopy vs. Conventional	9	291/1430	OR	1.343 (0.771, 2.340)	0.297	Yes	65.29%	No	No	No	NS	NA	Moderate
Zhao, 2016	Serum uric acid	Increased vs. decreased	4	932/1298	OR	1.370 (0.977, 1.923)	0.068	Yes	99.97%	No	Yes	No	NS	NA	Critically low
Pranata, 2020	Serum galectin-3 level	high vs. low	3	NR/180	HR	1.185 (0.988, 1.422)	0.067	Yes	75.03%	Yes	NA	Yes	NS	NA	Critically low
Aldaas 2020	HFpEF vs. HFrEF	HFpEF vs. HFrEF	6	436/1283	RR	1.015 (0.765, 1.346)	0.917	Yes	57.41%	No	No	Yes	NS	NA	Moderate
Peng, 2018	Statins	statins vs. control	10	545/1607	OR	0.808 (0.592, 1.103)	0.1796	Yes	40.77%	No	No	No	NS	NA	High
**Post-cardioversion**															
Yo 2014	High-sensitivity C-reactive protein(hs-CRP)	high hs-CRP vs. low hs-CRP	9	347/682	OR	6.119 (4.055, 9.232)	6.1 × 10^–18^	No	14.57%	Yes	Yes	Yes	IV	II	Critically low
Dentali 2011	statins	statins vs. control	12	785/1791	RR	0.777 (0.671, 0.900)	7.7 × 10^–4^	Yes	16.44%	No	No	No	IV	III	Critically low
Vitali 2019	CHA2DS2-VASc score	CHA2DS2-VASc ≥2 vs. CHA2DS2-VASc <2	3	89/478	OR	1.109 (1.049, 1.174)	3.0 × 10^–4^	Yes	0%	No	NP	Yes	IV	III	Critically low

AF, atrial fibrillation; BMI, body mass index; CA, catheter ablation; CE, class of evidence; CES, class of evidence sensitivity analysis; CHADS VASc, congestive heart failure, hypertension, age > 75 years, diabetes, stroke, vascular disease, age > 65, female Sex; CI, confidence interval; CPAP, continuous positive airway pressure; ES, effect size; DC, direct current; ESB, excess significance bias; HF, heart failure; HFpEF, heart failure with preserved ejection fraction; HFrEF, heart failure with reduced ejection fraction; hs-CRP, high sensitive C-reactive protein; HR, hazard ratio; i, indexed; I^2^, heterogeneity; K, number of studies for each factor; LA, left atrium; LAV, left atrial volume; LS, largest study with significant effect; MA, meta-analysis; *n*, number of cases; *N*, total number of cohorts per factor; NA, not assessable; NP, not pertinent because the number of observed studies is less than the expected; NR, not reported; OR, odds ratio; OSAS, obstructive sleep apnoea syndrome; PI, prediction interval; RR, risk ratio; SSEs, small study effects; RCT, randomized controlled trial.

In the meta-analyses of observational studies, 21 of the 26 associations examined (73%) had a nominally statistically significant effect (*P* ≤ 0.05) under the random-effects models, and four of these (15%) achieved a *P*-value <10^−6^. Sixteen associations (62%) had more than 1000 cases per association. Thirteen associations (50%) showed considerable heterogeneity (I^2^ > 50%), and 24 of 26 associations had a 95% prediction interval that excluded the null value. In 17 associations (65%), the ES of the largest study had a nominally statistically significant effect (*P* ≤ 0.05). Finally, small-study effects were found in seven associations (27%), and excess significance bias was found in eleven associations (42%).

When the credibility of evidence was assessed, no association presented convincing evidence. Only one association provided highly suggestive evidence (diagnosis of AF to ablation time), and two suggestive (hypertension and metabolic syndrome) (*Table 1*). The remaining 16 (76%) statistically significant associations between risk or protective factors and recurrence after CA of AF presented weak evidence (*Table 1*), while five associations were not significant (*Table 1*). Interestingly, when the criterion of associations with more than 1000 cases per study was excluded, three associations upgraded from low to highly suggestive level of evidence [burst-pacing post-CA, continuous positive airway pressure (CPAP) treatment in case of obstructive sleep apnoea syndrome (OSAS) and depression] and five more from a low to suggestive level of evidence, including high BMI and *P*-wave duration. (*Table 1*).

Only one meta-analysis of RCTs with two associations concerning protective factors of recurrence post-CA of AF was found. The analysis evaluated short-term AADs prior to CA of AF and the early and late recurrence risk. The quality of this meta-analysis was high according to AMSTAR2. It included six studies with 2655 participants (*Table 2*). The association between short-term AADs and late recurrence after CA of AF was non-significant, whereas the association between short-term antiarrhythmic use with the short time recurrence presented moderate GLE. No signs of heterogeneity or small study effects were found (*Table 2*).

**Table 2:** Predictors for AF recurrence, in meta-analyses of randomized controlled trials

Author, year	Predictor	Exposed/unexposed as in included MA	K	n/N	Metric	ES (95% CI)	*P*	PI include null value	I^2^	SSE	ESB	High RoB	GLE	AMSTAR 2 quality
**Post-cardioversion**														
Cao, 2012	Omega-3 fatty acids	Omega-3 fatty acids vs. control	6	455/759	OR	0.626 (0.348, 1.127)	0.118	Yes	66.43%	No	NP	>25	N/S	Critically low
Lafuente, 2015	Quinidine	Quinidine vs. control	7	1158/1624	OR	0.508 (0.401, 0.401)	0.000	No	0%	No	No	>25	Moderate	High
Lafuente, 2015	Disopyramide	Disopyramide vs. control	2	89/146	OR	0.525 (0.271, 1.019)	0.057	NA	0%	NA	NP	>25	N/S	High
Lafuente, 2015	Flecainide	Flecainide vs. control	4	276/511	OR	0.350 (0.200, 0.615)	0.0000	No	34.38%	No	No	>25	Moderate	High
Lafuente, 2015	Propafenone	Propafenone	5	652/1098	OR	0.366 (0.282, 0.476)	0.027	Yes	0%	No	NP	>25	Moderate	High
Lafuente, 2015	Class I others	Class I others vs. control	3	1138/1383	OR	0.718 (0.422, 1.221)	0.221	Yes	33.02%	No	NP	>25	N/S	High
Lafuente, 2015	Beta-blockers	Beta-blockers vs. control	2	375/562	OR	0.432 (0.143, 1.300)	0.135	NA	88.60%	NA	No	>25	Low	High
Lafuente, 2015	Amiodarone	Amiodarone vs. control	6	471/812	OR	0.189 (0.141, 0.253)	0.0000	No	0%	No	No	>25	Moderate	High
Lafuente, 2015	Azimilide	Azimilide vs. control	4	1260/1602	OR	0.702(0.549, 0.897)	0.0046	Yes	0%	No	No	>25	Moderate	High
Lafuente, 2015	Dofetilide	Dofetilide vs. control	3	811/1183	OR	0.338(0.193, 0.593)	0.0002	Yes	74.85%	Yes	NP	>25	Moderate	High
Lafuente, 2015	Dronedarone	Dronedarone vs. control	2	1001/1443	OR	0.585(0.459, 0.745)	0.0000	NA	0%	NA	NP	>25	Moderate	High
Lafuente, 2015	Sotalol	Sotalol vs. control	13	2189/2758	OR	0.512(0.404, 0.647)	0.0000	No	42.71%	No	Yes	>25	Moderate	High
Lafuente, 2015	Antiarrythmics	Use or not	51	9420/13 122	OR	0.433(0.374, 0.500)	0.0000	No	63.43%	Yes	No	>25	Moderate	High
Lafuente, 2015	Class Ia	Class Ia vs. control	8	1214/1716	OR	0.507(0.404, 0.637)	0.0000	No	0%	No	No	>25	Moderate	High
Lafuente, 2015	Class Ib	Class Ib vs. control	2	1092/1321	OR	0.845(0.631, 1.132)	0.259	NA	0%	NA	NP	>25	N/S	High
Lafuente, 2015	Class Ic	Class Ic vs. control	11	1010/1765	OR	0.357 (0.290, 0.441)	0.0000	No	0%	No	NP	>25	Moderate	High
Lafuente, 2015	Class I	Class I vs. control	21	3316/4802	OR	0.441(0.362, 0.538)	0.0000	No	40.61%	Yes	Yes	>25	Moderate	High
Lafuente, 2015	Class III	Class III vs. control	27	5693/8004	OR	0.428 (0.428, 0.529)	0.0000	Yes	72.12%	Yes	No	>25	Moderate	High
Lafuente, 2015	Disopyramide	Disopyramide vs. other Class I antiarrythmics	2	53/113	OR	0.755(0.358, 1.590)	0.459	NA	0%	NA	NP	>25	N/S	High
Lafuente, 2015	Amiodarone	Amiodarone vs. Class I drugs	5	371/643	OR	0.356(0.239, 0.532)	0.0000	Yes	31.41%	No	NP	>25	Moderate	High
Lafuente, 2015	Class III	Class III drugs vs. Class I drugs	13	1772/2875	OR	0.811(0.588, 1.119)	0.201	Yes	69.68%	No	NP	>25	N/S	High
Schneider 2010	RAAS	Use or not	8	990/3044	RR	0.53 (0.33, 0.85)	0.009	Yes	0%	No	Yes	N/R	Low	Critically Low
**Post-ablation**														
Chen 2016	Short-term Antiarrythmics (early recurrence of AF)	Use or not	6	1129/2667	RR	0.679 (0.526, 0.877)	0.003	Yes	58.38%	Yes	Yes	>25	Moderate	High
Chen 2016	Short-term Antiarrythmics (late recurrence of AF)	Use or not	6	849/ 2655	RR	0.917 (0.917, 1.023)	0.120	Yes	0%	No	NP	>25	N/S	High

CE, class of evidence; CI, confidence interval; ES, effect size; ESB, excess significance bias; GLE, GRADE level of evidence; GRADE, Grading of Recommendations Assessment, Development, and Evaluation; I^2^, heterogeneity; K, number of studies for each factor; LS, largest study with significant effect; *n*, number of cases; *N*, total number of cohort per factor; NA, not assessable; NP, not pertinent because the number of observed studies is less than the expected; NR, not reported; OR, odds ratio; PI, prediction interval; RoB, risk of bias; RR, risk ratio; SSE, small study effects.

### Risk and protective factors of recurrence after direct current cardioversion

Overall, only three meta-analyses of observational studies on risk and protective factors of recurrence after DC cardioversion were found. All of them were of low quality according to the AMSTAR2 scoring system (*Table 1* and Supplementary material online, *Appendix S5* in the Supplement). The median number of studies included in the meta-analyses was nine, with a median of 682 participants and 347 recurrences. All associations were statistically significant, with no evidence of heterogeneity (I^2^ < 30%). In one of three associations, the ES of the largest study had no statistically significant effect, and there was evidence of small-study effects. The prediction interval did not exclude the null value in two of three associations and there were signs of excess significance. All associations presented a low level of evidence. When the restriction of 1000 cases per association was withdrawn, the association of high-sensitive CRP (hs-CRP) with the risk of recurrence after DC cardioversion was upgraded to a highly suggestive level of evidence, and one of the statins and the CHA2DS2-VASc score were also suggestive (*Table 1*).

We found three meta-analyses of RCTs using 23 associations between protective factors and recurrence after DC cardioversion. The quality of the included meta-analyses according to the AMSTAR2 score was high in 20 meta-analyses of RCTs and low in only three. (*Table 2* and Supplementary material online, *Appendix S5*) The median number of studies included in meta-analyses of RCTs was 5.5 (IQR = 2.75–11.5), the median number of participants was 1430 (IQR = 730–2787), and the median number of cases was 1006 (IQR = 434–1388). Overall, 6 of 23 reported associations were non-significant (*P* < 0.05). Thirteen associations presented high heterogeneity (I^2^ > 50%), four showed small study effects, and three showed excess significance bias. When the RCT credibility criteria were applied, 14 associations between protective factors and recurrence of AF after CA of AF presented moderate GLE and one low. GLE (*Table 2*). These associations included using different AADs and RAAS blockers as protective factors against AF recurrence after DC cardioversion (*Table 2*).

## Discussion

To the best of our knowledge, this study is the first umbrella review of risk and protective factors for recurrence after CA and DC cardioversion of AF that assesses the levels of evidence of the published meta-analyses. A total of 53 risk and protective factors were examined, resulting in 39 significant associations between risk and protective factors and the risk of recurrence after CA or DC cardioversion of AF. However, no significant association was supported by convincing evidence.

Surprisingly, the association of established risk factors such as LA volume and female gender with recurrence of AF after CA was supported by only limited evidence. Other risk factors such as LA diameter and the use of AADs prior to CA were not assessed (using the including criteria of this meta-analysis) or were not statistically significant. These risk factors need to be re-evaluated by larger cohort studies and should not currently be considered to select patients with AF for CA.

The most important protective factor for AF recurrence after CA was the short duration (<1 year) between the time of AF diagnosis and AF ablation (RR: 0.73, 95% CI: 0.65–0.82).^15^ Previous studies have shown that the longer the AF persists, the more significant the risk of progressive atrial structural and electrical remodelling, and the higher the risk of unsuccessful CA.^15,32^ Implementing an early rhythm control therapy in patients with AF, either with AADs or CA, results in a reduced risk of adverse cardiovascular outcomes, including death from a cardiovascular cause.^33^

Other modifiable risk factors for AF recurrence after CA identified in the study were metabolic syndrome, obesity, and hypertension.^34^ Metabolic syndrome is defined as a combination of central obesity, hypertension, dyslipidemia, and glucose intolerance.^35^ The mechanism behind recurrence after CA and metabolic syndrome is not precise. However, metabolic syndrome has been associated with atrial conduction disturbances of conduction and refractoriness between the right and left atria,^36,37^ whereas obesity is associated with a shortened effective refractory period in the pulmonary veins.^38^ Furthermore, the use of CPAP in patients with OSAS, appears to protect them from AF recurrences after CA (RR: 0.59, 95% CI: 0.51–0.67). However, the level of evidence for this association was low due to the small number of of identified cases, which is why more extensive studies are required.

Depression was a highly significant (*P* = 1.8 × 10^−20^) risk factor for AF recurrence after CA (RR: 2.3, 95% CI: 1.75–2.9), supported by suggestive evidence.^39^ The mechanism of this association is unclear; however, it has been suggested that depression may be associated with chronic inflammation and can increase sympathetic activity, both factors that can mediate atrial modelling in patients with AF.^40,41^

Regarding procedural related predictors of recurrence of AF after CA ablation, non-inducibility of AF with burst-pacing was highly significant (*P* = 4.3 × 10^−11^) and was supported by highly suggestive evidence when the criterium of >1000 cases per association was not considered. Thus, larger studies are needed to confirm this association. Other factors, such as the use of adenosine to unveil reconnections in pulmonary veins after CA, proved to be non-significant. Regarding the risk of recurrence after DC cardioversion of AF, meta-analyses of RCTs showed that the use of AADs were significant protective factors supported by a moderate level of evidence. Several AADs, mainly belonging to pharmacologic classes IC and III, proved to maintain sinus rhythm after electrical cardioversion of AF. Depending on the drug, the recurrence of AF was reduced by 30 to 80% compared with controls. However, the success rate of DC cardioversion remained low, around 30% for the whole population.^42,43^ The level of evidence for these associations was moderate, mainly due to the high risk of bias of the original studies. This issue can be explained by the fact that most of the RCTs included in the original meta-analyses were implemented >10 years ago. The use of other drug categories such as b-blockers and omega-3 fatty acids also failed to achieve significance level.

In the current guidelines,^3^ CA is proposed as a safe and superior treatment option compared with AADs for rhythm control in patients with AF. However, it is pointed out that a more objective method of patient selection is needed. This umbrella review of meta-analyses is the first to provide data on the associations of various risk and protective factors with the risk of recurrence after CA of AF. The results of the study indicate the lack of associations with convincing evidence. Thus, no causality between meta-analyzed risk or protective factors and recurrence after CA or DC cardioversion can be reported. Larger cohort studies and RCTs are needed to improve the evidence base. The study also indicates that waiting time before implementing CA, obesity, and suffering from hypertension and metabolic syndrome or depression increase the risk of AF recurrence after CA. The use of burst pacing to check AF inducibility after CA can be considered. Finally, in patients undergoing DC cardioversion of AF, use AADs such as amiodarone and flecainide can decrease the risk of recurrence.

This umbrella review has several limitations. Initially, only meta-analyzed associations or meta-analyses with sufficient data were included. Therefore, other key factors might not have been considered, as they have not been evaluated in published meta-analyses. Second, the results from the primary analyses may have been affected by various unidentified covariates. Third, potentially meaningful subgroup analyses for different age groups, gender, and type of AF were not addressed. Finally, the grading system used can only provide indications of systematic biases but no proof of their nature and extent.

## Conclusions

This study reviewed and evaluated the literature of the epidemiological evidence for factors associated with recurrence of AF after CA and DC cardioversion. Although AF is a significant health issue, few risk and protective factors for AF recurrences have been identified, none of which supported by convincing evidence. An early CA strategy appears to reduce the risk of recurrence. Addressing comorbidities such as obesity, depression, metabolic syndrome, and hypertension prior to CA can increase the success of the intervention. The use of AADs, mainly amiodarone, flecainide, and propafenone, may increase the success rate of DC cardioversion. This study suggests that the factors with borderline significance and low level of evidence need to be analyzed further to achieve genuine associations. Further investigation of new predictors for successful CA procedures and DC cardioversions is needed.

## Supplementary material

Supplementary material is available at *Europace* online.

## Supplementary Material

euac143_Supplementary_Data

## Data Availability

The data sets used and/or analyzed during the present study are available from the corresponding author on reasonable request.
