# Supplementary material for: Predictors of recurrence after catheter ablation and electrical cardioversion of atrial fibrillation: an umbrella review of meta-analyses
Source: Europace. 2022 Aug 29;25(1):40–8. doi: 10.1093/europace/euac143 (PMC10103559; doi:10.1093/europace/euac143)
Supplement: euac143_Supplementary_Data [file euac143_supplementary_data.zip › revised manuscript_highlighted changes.docx]

Predictors of recurrence after catheter ablation and electrical cardioversion of atrial fibrillation: an umbrella review of meta-analyses

Emmanouil Charitakis^1^, Elena Dragioti^2^, Maria Stratinaki^3^, Dafni Korela^3^, Stylianos Tzeis^4^, Henrik Almroth^1^, Ioan Liuba^1^, Anders Hassel Jönsson^1^, Georgios Charalambous^5^, Lars O Karlsson^1^, Dimitrios Tsartsalis^5^.

1. Department of Cardiology and Department of Health, Medicine and Caring Sciences, Linköping University, Linköping, Sweden
2. Pain and Rehabilitation Centre and Department of Health, Medicine and Caring Sciences, Linköping University, Linköping, Sweden
3. Department of Cardiology, Venizeleio General Hospital, Heraklion, Crete, Greece
4. Mitera Hospital, Hygeia Group, Athens Greece
5. Department of Emergency Medicine, 'Hippokration' Hospital, Athens, Greece Athens, Greece.

**Address for Correspondence:**

Dimitrios Tsartsalis, MD, PhD, FESC

Department of Emergency Medicine 'Hippokration' Hospital,

Vasilissis Sofias 114, 11527 Athens, Greece.

Telephone: +306974791798/FAX:+302114135469

E-mail : [dtsartsalis@gmail.com](mailto:dtsartsalis@gmail.com)

ORCID ID: 0000-0002-7535-938X

**Word count: 3346**

**Tables: 2**

**Figures:1**

# Abstract

**Aim:** The recurrence rates after catheter ablation (CA) and direct current (DC) cardioversion remain high, although they have been established treatments of rhythm control of atrial fibrillation (AF). This umbrella review systematically appraises published meta-analyses of both observational and randomized controlled trials (RCT) for the association of risk and protective factors for arrhythmia recurrence after CA and DC-cardioversion of AF.

**Methods:** Three bibliographic databases were searched up to June 2021. Evidence of association was rated as convincing, highly suggestive, suggestive, weak, or not significant with respect to observational studies and as high, moderate, low, or very low with respect to RCTs, according to established criteria.

**Results**: Thirty-one meta-analyses were included. Of the 28 associations between CA and the risk of arrhythmia recurrence, none presented convincing evidence, and only the time from diagnosis to ablation (DTA) over one year provided highly suggestive evidence. The association between hypertension and metabolic profile provided suggestive evidence. The associations of class IC and III antiarrhythmic drugs use with the recurrence after DC-cardioversion were supported by an intermediate level of evidence

**Conclusions:** Although AF is a major health issue, few risk- and protective factors for AF recurrence have been identified. None of these factors examined were supported by convincing evidence, while established factors like female gender and left atrial volume showed only weak association. An early CA strategy combined with treatment of metabolic syndrome and hypertension prior to CA may reduce the risk of arrhythmia recurrence. The use of antiarrhythmics can increase the success rate of DC-cardioversion.

**Keywords:** catheter ablation, atrial fibrillation, umbrella review, electrical cardioversion

**Systematic review registration**: PROSPERO registry number: CRD42021270613

# What’s new

- Our study is the first umbrella review to systematically appraise published meta-analyses of both observational and randomized controlled trials for the association of risk and protective factors for arrhythmia recurrence after catheter ablation (CA) and direct current (DC) cardioversion of atrial fibrillation (AF).
- Few risk- and protective factors of arrhythmia recurrence after catheter ablation (CA) and direct current (DC) cardioversion of AF have been identified.
- None of the factors examined showed association with AF recurrence supported by convincing evidence.
- An early CA strategy and management of metabolic syndrome and hypertension prior to CA can reduce the risk of arrhythmia recurrence.
- The use of antiarrhythmic drugs, mainly amiodarone, flecainide, and propafenone, can increase the success rate of DC-cardioversion.

# Introduction

The prevalence of atrial fibrillation (AF) among adults over 55 years of age in the European Union was estimated at approximately 8.8 million in 2010. An increase to around 14 million is forecast by 2060 (1). These numbers are influenced by both unmodifiable factors such as advancing age and modifiable risk factors associated with modern lifestyle such as high body mass index (BMI), physical inactivity, hypertension, obstructive sleep apnea syndrome (OSAS) and psychosocial stress (2). Atrial fibrillation is associated with an increased risk of death, primarily from heart failure (HF) and stroke (3).

Currently, stroke prevention with oral anticoagulation has reduced the risk of death in patients with AF. However, many patients remain symptomatic as well the risk of HF (4).

Synchronized direct current (DC) cardioversion is an integral part of rhythm control in patients with AF (5). Direct cardioversion terminates AF in over 90% of cases with rare complications (6). Nevertheless, the recurrence rate of AF is high, approximately 50% in the first two weeks and 65% in the first year after cardioversion (7). Consequently, various alternatives such as the ‘wait and see’ approach in patients with recent-onset AF and the use of hybrid treatment, combining DC-cardioversion with pretreatment with antiarrhythmic drugs (AADs) have been proposed (5, 7). Identifying predictors of successful DC-cardioversion is crucial. So far, age, functional class of the patients, and eventual prior treatment with AAD have been identified as predictors of successful cardioversion (6).

Catheter ablation (CA) is an established and safe treatment option for rhythm control in patients with symptomatic AF (3, 8). Pulmonary vein isolation (PVI) has been the basis of CA procedures for AF since the pioneering observation of induction of AF by ectopic beats from the pulmonary veins (9, 10). CA has evolved substantially over the past few decades, with several different tools becoming accessible and different approaches being adopted. To date, none of the novel approaches have been proven superior to PVI alone (11). Success rates vary between 50% and 80%, depending on the type of AF (paroxysmal or persistent) (11, 12). The likelihood of successful CA is related to both procedure and patient characteristics (11, 13). Given the moderate success rate of CA in patients with AF, several studies have explored different factors such as gender, AF duration, age, as predictors of recurrence post-CA of AF (14-16). No study has yet collectively summarized, evaluated, and graded the evidence of the clinically applicable research on this topic.

The aim of this paper is to summarize the existing evidence on risk and protective factors associated with the risk of recurrence after CA and DC-cardioversion of AF across published meta-analyses through an umbrella review. Following best research practices, the evidence from the available meta-analyses on this topic was ranked based on sample size, the strength of the association, and the presence of various biases (17, 18).

# Methods

The Preferred Reporting Items for Systematic Reviews and Meta-Analyses (PRISMA) (19) reporting guidelines and the Meta-Analysis of Observational Studies in Epidemiology (MOOSE) guidelines (20) (Appendix 1 in the Supplement) were used in this study. The study protocol was registered in the PROSPERO database. (CRD42021270613).

## **Data Selection, Search Strategy, and Selection Criteria**

Bibliographic databases (PubMed, Web of Science, and the Cochrane Database of Systematic Reviews) were searched up to May 28, 2021, to identify systematic reviews with meta-analysis of observational or randomized controlled trials (RCT) that examined associations between non-genetic risk or protective factors, with the risk of recurrence after CA or/and DC-cardioversion of AF as a primary or secondary endpoint. Our search strategy was broad to identify all eligible studies using terms related to AF and meta-analysis (Appendix 2 in the Supplement). The reference lists of eligible studies were also examined to identify additional studies.

Two researchers (DK, MS) independently conducted the initial search for relevant articles. The full texts of the retrieved articles were further examined for eligibility by the same researchers. Records of any discrepancies regarding the eligibility for inclusion were reviewed by a third investigator (EC), and disagreements were resolved through discussion.

We included only peer-reviewed meta-analyses of RCTs or observational studies with either a cohort, case-control, or nested case-control study design and examined any association between recurrence of AF after CA or DC-cardioversion and risk- or protective factors. In the case of multiple meta-analyses evaluating the same risk and/or protective factors, we kept the meta-analysis with the largest number of included studies (21). All reported outcomes for eligible meta-analyses were considered for inclusion.

Meta-analyses were excluded for 1) study designs other than those mentioned above (cross-sectional, letter to the editor); 2) a non-systematic selection of observational studies or RCTs and non-systematic reviews; 3) examining genetic variants as risk factors for recurrence after CA or DC-cardioversion; 4) studies published in languages other than English; 5) insufficient data for quantitative synthesis were provided; or 6) they presented study-specific effects estimates other than odds ratio (OR), hazard ratio (HR), or relative risk (RR), i.e., mean difference. Reasons for exclusion after full-text assessment were listed in the supplementary material (Appendix 3 in the Supplement).

## **Data Extraction**

Two researchers (DK, MS) independently performed data extraction from each eligible article using a predefined extraction form (EXCEL 365). Any disagreements were resolved through discussion. For each meta-analysis, the collected variables included: first authors’ name, year of publication, journal, standard identifier (DOI), number of primary studies, total sample size, and risk or protective factors assessed. For each primary study, we collected: the first authors´ name, year of publication, study design, sample size (exposure and non-exposure), and relative risk estimates (i.e., HR, OR, RR) with the subsequent 95% confidence interval (CI).

## **Quality assessment**

Risk of bias (RoB) was assessed using the AMSTAR2 tool (A Measurement Tool to Assess systematic Reviews, available at <https://amstar.ca/Amstar-2.php>)https://amstar.ca/Amstar-2.php. This tool was designed to assess meta-analyses of both randomized and non-randomized studies and applies criteria within ten original domains. The RoB assessment was performed by two reviewers (DT, MS) and checked by two others (EC, DT) (22).

## **Data Synthesis and Analysis**

The effect size (ES) of different studies reported in each meta-analysis was extracted for each association. We re-measured the pooled effect sizes and 95% CIs employing random-effects models (23) and examined inter-study heterogeneity using the I2 statistic (24). The small-study effect bias was evaluated with the Egger regression asymmetry test and random-effects summary effect size to determine whether smaller studies produced larger effect sizes compared to larger studies (25, 26). Finally, the excess significance bias was measured to decide whether the observed number of studies with statistically significant results differed from the anticipated number of studies with statistically significant results (27). The anticipated number of statistically significant studies per association was calculated by summing the statistical power estimates for each component study. The power estimates of each component study depend on the reasonable ES for the examined association, which are assumed to be the ES of the largest study (i.e., the smallest standard error) per association. A p-value ≤ 0.10 was considered statistically significant for excess significance bias( 27). All analyses were performed using Stata 17.0 (StataCorp, College Station, TX) and R v.4.0.3 (The R Foundation for Statistical Computing, Auckland, NZ).

Suitable associations from observational studies were categorized into five classes, according to the power of the evidence of possible risk or protective factors: convincing (class I), highly suggestive (class II), suggestive (class III), weak (class IV), and not significant (NS) (eTable 1, Appendix 4 in the Supplement), consistent with previous umbrella reviews (28).

Regarding RCTs, the credibility of evidence was categorized according to the summary effect (p-value < 0.01, 0.01 ≤ p-value < 0.05, p-value ≥ 0.05), 95% prediction interval (excluding the null or not), presence of large heterogeneity (I² > 50%), small study effects (p < 0.10), and excess significance (p < 0.10) (29). Additionally, GRADE (Grading of Recommendations Assessment, Development, and Evaluation) levels of evidence (GLE) using a modified concrete set of rules was also employed as previously described (30, 31). Four areas were evaluated: 1) imprecision, by the number of participants in the pooled analysis (if 100-199 participants, GLE was downgraded by 1 level; if <100 participants, downgraded by two levels); 2) RoB original study quality, by the proportion of RCTs included in the pooled analysis with low RoB for randomization and observer blinding (if ≥ 75% of RCTs had low RoB or RoB not reported, GLE was downgraded by 1 level); 3) inconsistency, by heterogeneity (if I² > 75%, downgraded by 1 level); and 4) RoB review quality, by the responses to AMSTAR2 questionnaire (if moderate quality, downgraded by 1 level; if low or critically low quality, downgraded by two levels). Then, reviews were classified as high, moderate, low, or very low according to GLE (eTable 2, Appendix 4 in the Supplement).

# Results

## **Literature Search**

The initial search yielded 4179 publications. After title and abstract assessment, 96 eligible articles were identified. Then 65 articles were excluded after a full-text review (Appendix 3), and 31 articles were included for analysis. Twenty-six studies evaluated risk and protective factors for recurrence of AF post-CA and reported 28 associations, while six studies assessed risk- and protective factors for AF recurrence after DC-cardioversion and reported 25 associations (Figure 1). Background factors of the included studies are presented in Appendix 5 in the supplement.

## **Risk and protective factors of recurrence post-CA of AF**

The quality score of the meta-analyses of observational studies on risk and protective factors for recurrence of AF post-CA, following AMSTAR2, was low and critically low in 14 studies, moderate in six and high in six (Table 1 and Appendix 5 in the Supplement). The median number of studies included in the original meta-analyses was 6 (IQR = 5 – 10), the median number of patients was 1,304 (IQR = 639 – 3,806), and the median number of incidences of recurrence was 454 (IQR = 291 – 932).

In the meta-analyses of observational studies, 21 of the 26 associations examined (73%) had a nominally statistically significant effect (p ≤ 0.05) under the random-effects models, and 4 of these (15%) achieved a p-value <10^-6^. Sixteen associations (62%) had more than 1,000 cases per association. Thirteen associations (50%) showed considerable heterogeneity (I^2^ > 50%), and 24 of 26 associations had a 95% prediction interval that excluded the null value. In 17 associations (65%), the ES of the largest study had a nominally statistically significant effect (p ≤ 0.05). Finally, small-study effects were found in seven associations (27%), and excess significance bias was found in eleven associations (42%).

When the credibility of evidence was assessed, no association presented convincing evidence. Only one association provided highly suggestive evidence (diagnosis of AF to ablation time), and two suggestive (hypertension and metabolic syndrome) (Table 1). The remaining 16 (76%) statistically significant associations between risk or protective factors and recurrence after CA of AF presented weak evidence (Table 1), while five associations were not significant (Table 1). Interestingly, when the criterion of associations with more than 1,000 cases per study was excluded, three associations upgraded from low to highly suggestive level of evidence (burst-pacing post-CA, continuous positive airway pressure (CPAP) treatment in case of obstructive sleep apnoea syndrome (OSAS) and depression) and five more from a low to suggestive level of evidence, including high body mass index (BMI) and p-wave duration. (Table 1).

Only one meta-analysis of RCTs with two associations concerning protective factors of recurrence post-CA of AF was found. The analysis evaluated short-term AADs prior to CA of AF and the early and late recurrence risk. The quality of this meta-analysis was high according to AMSTAR2. It included six studies with 2655 participants (Table 2). The association between short-term AADs and late recurrence after CA of AF was non-significant, while the association between short-term antiarrhythmic use with the short time recurrence presented moderate GLE. No signs of heterogeneity or small study effects were found (Table 2).

## **Risk and protective factors of recurrence after DC-cardioversion**

Overall, only three meta-analyses of observational studies on risk and protective factors of recurrence after DC-cardioversion were found. All of them were of low quality according to the AMSTAR2 scoring system (Table 1 and Appendix 5 in the Supplement). The median number of studies included in the meta-analyses was 9, with a median of 682 participants and 347 recurrences. All associations were statistically significant, with no evidence of heterogeneity (I^2^<30%). In one of three associations, the ES of the largest study had no statistically significant effect, and there was evidence of small-study effects. The prediction interval did not exclude the null value in two of three associations and there were signs of excess significance. All associations presented a low level of evidence. When the restriction of 1000 cases per association was withdrawn, the association of high-sensitive CRP (hsCRP) with the risk of recurrence after DC-cardioversion was upgraded to a highly suggestive level of evidence, and one of the statins and the CHA2DS2 VASc score were also suggestive (Table 1).

We found three meta-analyses of RCTs employing 23 associations between protective factors and recurrence after DC-cardioversion. The quality of the included meta-analyses according to the AMSTAR2 score was high in 20 meta-analyses of RCTs and low in only three. (Table 2 and Appendix 5) The median number of studies included in meta-analyses of RCTs was 5.5 (IQR=2.75-11.5), the median number of participants was 1430 (IQR=730-2787), and the median number of cases was 1006 (IQR=434-1388). Overall, 6 of 23 reported associations were non-significant (p < 0.05). Thirteen associations presented high heterogeneity (I^2^ > 50%), four showed small study effects, and three showed excess significance bias. When the RCT credibility criteria were applied, 14 associations between protective factors and recurrence of AF after CA of AF presented moderate GLE and one low. GLE (Table 2). These associations included using different AADs and RAAS blockers as protective factors against AF recurrence after DC-cardioversion (Table 2).

## Discussion

To the best of our knowledge, this study is the first umbrella review of risk and protective factors for recurrence after CA and DC-cardioversion of AF that assesses the levels of evidence of the published meta-analyses. A total of 53 risk and protective factors were examined, resulting in 39 significant associations between risk and protective factors and the risk of recurrence after CA or DC-cardioversion of AF. However, no significant association was supported by convincing evidence.

Surprisingly, the association of established risk factors such as LA volume and female gender with recurrence of AF after CA ~~of~~ was supported by only limited evidence. Other risk factors such as LA diameter and the use of AADs prior to CA were not assessed (using the including criteria of this meta-analysis) or were not statistically significant. These risk factors need to be reevaluated by larger cohort studies and should not currently be considered to select patients with AF for CA.

The most important protective factor for AF recurrence after CA was the short duration (<1year) between the time of AF diagnosis and AF ablation (RR: 0.73, CI 95 %: 0.65-0.82)(15). Previous studies have shown that the longer the atrial fibrillation persists, the more significant the risk of progressive atrial structural and electrical remodeling, and the higher the risk of unsuccessful CA (15, 32). Implementing an early rhythm control therapy in patients with AF, either with AADs or CA, results in a reduced risk of adverse cardiovascular outcomes, including death from a cardiovascular cause (33).

Other modifiable risk factors for AF recurrence after CA identified in the study were metabolic syndrome, obesity, and hypertension (34). Metabolic syndrome is defined as a combination of central obesity, hypertension, dyslipidemia, and glucose intolerance (35). The mechanism behind recurrence after CA and metabolic syndrome is not precise. However, metabolic syndrome has been associated with atrial conduction disturbances of conduction and refractoriness between the right and left atria (36, 37), while obesity is associated with a shortened effective refractory period in the pulmonary veins (38). Furthermore, the use of CPAP in patients with OSAS, appears to protect them from AF recurrences after CA (RR: 0.59, CI 95%: 0.51-0.67). However, the level of evidence for this association was low due to the small number of of identified cases , which is why more extensive studies are required.

Depression was a highly significant (p=1.8x10^-20^) risk factor for AF recurrence after CA (RR:2.3, Ci 95%:1.75-2.9), supported by suggestive evidence (39). The mechanism of this association is unclear; however, it has been suggested that depression may be associated with chronic inflammation and can increase sympathetic activity, both factors that can mediate atrial modeling in patients with AF (40, 41).

Regarding procedural related predictors of recurrence of AF after CA-ablation, non-inducibility of AF with burst-pacing was highly significant (p=4.3x10^-11^) and was supported by highly suggestive evidence when the criterium of >1000 cases per association was not considered. Thus, larger studies are needed to confirm this association. Other factors, such as the use of adenosine to unveil reconnections in pulmonary veins after CA, proved to be non-significant. Regarding the risk of recurrence after DC-cardioversion of AF, meta-analyses of RCTs showed that the use of AADs were significant protective factors supported by a moderate level of evidence. Several AADs, mainly belonging to pharmacologic classes IC and III, proved to maintain sinus rhythm after electrical cardioversion of AF. Depending on the drug, the recurrence of AF was reduced by 30% to 80% compared to controls. However, the success rate of DC-cardioversion remained low, around 30% for the whole population (42, 43). The level of evidence for these associations was moderate, mainly due to the high risk of bias of the original studies. This issue can be explained by the fact that most of the RCTs included in the original meta-analyses were implemented more than ten years ago. The use of other drug categories such as b-blockers and omega-3 fatty acids also failed to achieve significance level.

In the current guidelines (3), CA is proposed as a safe and superior treatment option compared to AADs for rhythm control in patients with AF. However, it is pointed out that a more objective method of patient selection is needed. This umbrella review of meta-analyses is the first to provide data on the associations of various risk and protective factors with the risk of recurrence after CA of AF. The results of the study indicate the lack of associations with convincing evidence. Thus, no causality between meta-analyzed risk or protective factors and recurrence after CA or DC-cardioversion can be reported. Larger cohort studies and RCTs are needed to improve the evidence base. The study also indicates that waiting time before implementing CA, obesity, and suffering from hypertension and metabolic syndrome or depression increase the risk of AF recurrence after CA. The use of burst pacing to check AF-inducibility after CA can be considered. Finally, in patients undergoing DC-cardioversion of AF, use AADs such as amiodarone and flecainide can decrease the risk of recurrence.

This umbrella review has several limitations. Initially, only meta-analyzed associations or meta-analyses with sufficient data were included. Therefore, other key factors might not have been considered , as they have not been evaluated in published meta-analyses. Second, the results from the primary analyses may have been affected by various unidentified covariates. Third, potentially meaningful subgroup analyses for different age groups, gender, and type of AF were not addressed. Finally, the grading system used can only provide indications of systematic biases but no proof of their nature and extent.

# Conclusions

This study reviewed and evaluated the literature of the epidemiological evidence for factors associated with recurrence of atrial fibrillation after CA and DC-cardioversion. Although AF is a significant health issue, few risk and protective factors for AF recurrences have been identified, none of which supported by convincing evidence. An early CA strategy appears to reduce the risk of recurrence. Addressing comorbidities such as obesity, depression, metabolic syndrome, and hypertension prior to CA can increase the success of the intervention. The use of AADs, mainly amiodarone, flecainide, and propafenone, may increase the success rate of DC-cardioversion. This study suggests that the factors with borderline significance and low level of evidence need to be analyzed further to achieve genuine associations. Further investigation of new predictors for successful CA procedures and DC-cardioversions is needed.

Funding sources

None.

# Conflict of interest

None declared.

**Availability of data and materials**

The datasets used and/or analyzed during the current study are available from the corresponding author on reasonable request.

# References

1. Krijthe BP, Kunst A, Benjamin EJ, Lip GY, Franco OH, Hofman A, et al. Projections on the number of individuals with atrial fibrillation in the European Union, from 2000 to 2060. Eur Heart J 2013;34:2746-51.

2. Kornej J, Börschel CS, Benjamin EJ, Schnabel RB. Epidemiology of Atrial Fibrillation in the 21st Century: Novel Methods and New Insights. Circ Res 2020;127:4-20.

3. Hindricks G, Potpara T, Dagres N, Arbelo E, Bax JJ, Blomstrom-Lundqvist C, et al. 2020 ESC Guidelines for the diagnosis and management of atrial fibrillation developed in collaboration with the European Association of Cardio-Thoracic Surgery (EACTS). Eur Heart J. 2020;42:373-498.

4. Kotecha D, Piccini JP. Atrial fibrillation in heart failure: what should we do? Eur Heart J 2015;36:3250-7.

5. Pluymaekers NAHA, Dudink EAMP, Luermans JGLM, Meeder JG, Lenderink T, Widdershoven J, et al. Early or Delayed Cardioversion in Recent-Onset Atrial Fibrillation. N Engl J Med 2019;380:1499-508.

6. Brandes A, Crijns H, Rienstra M, Kirchhof P, Grove EL, Pedersen KB, et al. Cardioversion of atrial fibrillation and atrial flutter revisited: current evidence and practical guidance for a common procedure. Europace 2020;22:1149-61.

7. Van Gelder IC, Tuinenburg AE, Schoonderwoerd BS, Tieleman RG, Crijns HJ. Pharmacologic versus direct-current electrical cardioversion of atrial flutter and fibrillation. Am J Cardiol 1999;84:147r-51r.

8. Loring Z, Holmes DN, Matsouaka RA, Curtis AB, Day JD, Desai N, et al. Procedural Patterns and Safety of Atrial Fibrillation Ablation: Findings From Get With The Guidelines-Atrial Fibrillation. Circ Arrhythm Electrophysiol 2020;13:e007944.

9. Haissaguerre M, Jais P, Shah DC, Takahashi A, Hocini M, Quiniou G, et al. Spontaneous initiation of atrial fibrillation by ectopic beats originating in the pulmonary veins. N Engl J Med 1998;339:659-66.

10. Calkins H, Hindricks G, Cappato R, Kim YH, Saad EB, Aguinaga L, et al. 2017 HRS/EHRA/ECAS/APHRS/SOLAECE expert consensus statement on catheter and surgical ablation of atrial fibrillation: Executive summary. Heart Rhythm 2017;14:e445-e94.

11. Verma A, Jiang CY, Betts TR, Chen J, Deisenhofer I, Mantovan R, et al. Approaches to catheter ablation for persistent atrial fibrillation. N Engl J Med 2015;372:1812-22.

12. Ganesan AN, Shipp NJ, Brooks AG, Kuklik P, Lau DH, Lim HS, et al. Long-term outcomes of catheter ablation of atrial fibrillation: a systematic review and meta-analysis. J Am Heart Assoc 2013;2:e004549.

13. Link MS, Haïssaguerre M, Natale A. Ablation of Atrial Fibrillation. Circulation 2016;134:339-52.

14. Vallakati A, Reddy M, Sharma A, Kanmanthareddy A, Sridhar A, Pillarisetti J, et al. Impact of gender on outcomes after atrial fibrillation ablation. Int J Cardiol 2015;187:12-6.

15. Chew DS, Black-Maier E, Loring Z, Noseworthy PA, Packer DL, Exner DV, et al. Diagnosis-to-Ablation Time and Recurrence of Atrial Fibrillation Following Catheter Ablation: A Systematic Review and Meta-Analysis of Observational Studies. Circ Arrhythm Electrophysiol 2020;13:e008128.

16. Kornej J, Hindricks G, Shoemaker MB, Husser D, Arya A, Sommer P, et al. The APPLE score: a novel and simple score for the prediction of rhythm outcomes after catheter ablation of atrial fibrillation. Clin Res Cardiol 2015;104:871-6.

17. Ioannidis J. Next-generation systematic reviews: prospective meta-analysis, individual-level data, networks and umbrella reviews. Br J Sports Med 2017;51:1456-8.

18. Ioannidis JP. Integration of evidence from multiple meta-analyses: a primer on umbrella reviews, treatment networks and multiple treatments meta-analyses. CMAJ 2009;181:488-93.

19. Moher D, Liberati A, Tetzlaff J, Altman DG. Preferred reporting items for systematic reviews and meta-analyses: the PRISMA statement. PLoS Med 2009;6:e1000097.

20. Stroup DF, Berlin JA, Morton SC, Olkin I, Williamson GD, Rennie D, et al. Meta-analysis of observational studies in epidemiology: a proposal for reporting. Meta-analysis Of Observational Studies in Epidemiology (MOOSE) group. JAMA 2000;283:2008-12.

21. Raglan O, Kalliala I, Markozannes G, Cividini S, Gunter MJ, Nautiyal J, et al. Risk factors for endometrial cancer: An umbrella review of the literature. Int J Cancer 2019;145:1719-30.

22. Shea BJ, Reeves BC, Wells G, Thuku M, Hamel C, Moran J, et al. AMSTAR 2: a critical appraisal tool for systematic reviews that include randomised or non-randomised studies of healthcare interventions, or both. BMJ 2017;358:j4008.

23. DerSimonian R, Laird N. Meta-analysis in clinical trials. Control Clin Trials 1986;7:177-88.

24. Higgins JP, Thompson SG, Deeks JJ, Altman DG. Measuring inconsistency in meta-analyses. BMJ 2003;327:557-60.

25. Dragioti E, Karathanos V, Gerdle B, Evangelou E. Does psychotherapy work? An umbrella review of meta-analyses of randomized controlled trials. Acta psychiatr Scand 2017;136:236-46.

26. Dragioti E, Evangelou E, Larsson B, Gerdle B. Effectiveness of multidisciplinary programmes for clinical pain conditions: An umbrella review. J Rehabil Med 2018;50:779-91.

27. Ioannidis JP, Trikalinos TA. An exploratory test for an excess of significant findings. Clin Trials. 2007;4:245-53.

28. Belbasis L, Mavrogiannis MC, Emfietzoglou M, Evangelou E. Environmental factors, serum biomarkers and risk of atrial fibrillation: an exposure-wide umbrella review of meta-analyses. Eur J Epidemiol 2020;35:223-39.

29. Li X, Meng X, Timofeeva M, Tzoulaki I, Tsilidis KK, Ioannidis JP, et al. Serum uric acid levels and multiple health outcomes: umbrella review of evidence from observational studies, randomised controlled trials, and Mendelian randomisation studies. BMJ 2017;357:j2376.

30. Schünemann H BJ, Guyatt G, Oxman A, eds. GRADE handbook for grading quality of evidence and strength of recommendations 2013. https://gdt.gradepro.org/app/handbook/handbook.html (updated October 2013)

31. Pollock A, Farmer SE, Brady MC, Langhorne P, Mead GE, Mehrholz J, et al. An algorithm was developed to assign GRADE levels of evidence to comparisons within systematic reviews. J Clin Epidemiol 2016;70:106-10.

32. Verma A, Wazni OM, Marrouche NF, Martin DO, Kilicaslan F, Minor S, et al. Pre-existent left atrial scarring in patients undergoing pulmonary vein antrum isolation: an independent predictor of procedural failure. J Am Coll Cardiol 2005;45:285-92.

33. Kirchhof P, Camm AJ, Goette A, Brandes A, Eckardt L, Elvan A, et al. Early Rhythm-Control Therapy in Patients with Atrial Fibrillation. N Engl J Med 2020;383:1305-16.

34. Lin KJ, Cho SI, Tiwari N, Bergman M, Kizer JR, Palma EC, et al. Impact of metabolic syndrome on the risk of atrial fibrillation recurrence after catheter ablation: systematic review and meta-analysis. J Interv Card Electrophysiol 2014;39:211-23.

35. Alberti KG, Eckel RH, Grundy SM, Zimmet PZ, Cleeman JI, Donato KA, et al. Harmonizing the metabolic syndrome: a joint interim statement of the International Diabetes Federation Task Force on Epidemiology and Prevention; National Heart, Lung, and Blood Institute; American Heart Association; World Heart Federation; International Atherosclerosis Society; and International Association for the Study of Obesity. Circulation 2009;120:1640-5.

36. Chang SL, Tuan TC, Tai CT, Lin YJ, Lo LW, Hu YF, et al. Comparison of outcome in catheter ablation of atrial fibrillation in patients with versus without the metabolic syndrome. Am J Cardiol 2009;103:67-72.

37. Tanaka K, Zlochiver S, Vikstrom KL, Yamazaki M, Moreno J, Klos M, et al. Spatial Distribution of Fibrosis Governs Fibrillation Wave Dynamics in the Posterior Left Atrium During Heart Failure. Circ Res 2007;101:839-47.

38. Munger TM, Dong YX, Masaki M, Oh JK, Mankad SV, Borlaug BA, et al. Electrophysiological and hemodynamic characteristics associated with obesity in patients with atrial fibrillation. J Am Coll Cardiol 2012;60:851-60.

39. Zhuo C, Ji F, Lin X, Jiang D, Wang L, Tian H, et al. Depression and recurrence of atrial fibrillation after catheter ablation: a meta-analysis of cohort studies. J Affect Disord 2020;271:27-32.

40. Valkanova V, Ebmeier KP, Allan CL. CRP, IL-6 and depression: A systematic review and meta-analysis of longitudinal studies. J Affect Disord 2013;150:736-44.

41. Shi S, Liang J, Liu T, Yuan X, Ruan B, Sun L, et al. Depression Increases Sympathetic Activity and Exacerbates Myocardial Remodeling after Myocardial Infarction: Evidence from an Animal Experiment. PLoS One 2014;9:e101734.

42. Lafuente-Lafuente C, Valembois L, Bergmann JF, Belmin J. Antiarrhythmics for maintaining sinus rhythm after cardioversion of atrial fibrillation. Cochrane Database Syst Rev 2015;3:CD005049 pub4: 1-155

43. Valembois L, Audureau E, Takeda A, Jarzebowski W, Belmin J, Lafuente-Lafuente C. Antiarrhythmics for maintaining sinus rhythm after cardioversion of atrial fibrillation. Cochrane Database Syst Rev 2019;9:CD005049 pub5.:1-200

Figure legends

Figure 1: Flowchart of the study selection process

# Tables

# Table 1. Predictors for AF recurrence, in meta-analyses of observational studies.

| **Author, Year** | **predictor** | | **Exposed / Unexposed as in included MA** | | **K** | | **n / N** | | **Metric** | | **ES (95% CI)** | | **p** | | **PI include null value** | | **I^2^** | | **SSE** | | **ESB** | | **LS sign** | | **CE** | | **CES2 (n>1,000)** | | **AMSTAR 2 Quality** | |  |
| --- | --- | --- | --- | --- | --- | --- | --- | --- | --- | --- | --- | --- | --- | --- | --- | --- | --- | --- | --- | --- | --- | --- | --- | --- | --- | --- | --- | --- | --- | --- | --- |
| **Post ablation** | | | | | | | | | | | | | | | | | | | | | | | | | | | | | | |  |
| Chew , 2020 | Diagnosis-to-Ablation Time (DAT) | | DAT ≤ 1 year vs DAT > 1 year | | 6 | | 2220/ 4950 | | RR | | 0.729 ( 0.646 , 0.822 ) | | 2.7x10-7 | | Yes | | 53.73% | | No | | NP | | Yes | | II | | II | | High | |  |
| Lin 2013 | metabolic syndrome | | metabolic syndrome vs no metabolic syndrome | | 5 | | 1184/3320 | | RR | | 1.63  (1.25, 2.12) | | 2.9x10-4 | | Yes | | 54.3 | | Yes | | Yes | | Yes | | III | | III | | Low | |  |
| Lin 2013 | | hypertension | | Hypertension vs no hypertension | | 17 | | 3249/11430 | | RR | | 1.31  (1.13, 1.51) | | 2.6x10-4 | | Yes | | 47.5 | | No | | Yes | | No | | III | | III | | Low | |
| Liu 2020 | Burst pacing- post CA | | AF inducibility vs AF noninducibility | | 14 | | 702/2628 | | RR | | 0.661  (0.585, 0.748) | | 4.3x10-11 | | Yes | | 0% | | No | | Yes | | Yes | | IV | | II | | Critically low | |  |
| Zhuo 2020 | depression | | depression vs no depression | | 7 | | 306/985 | | RR | | 2.245  (1.750, 2.879) | | 1.8x10-20 | | No | | 29.17% | | Yes | | Yes | | Yes | | IV | | II | | Critically low | |  |
| Shukla, 2015 | OSAS + CPAP | | CPAP vs no CPAP | | 7 | | 494 /1087 | | RR | | 0.585 ( 0.511 0.670 ) | | 1.7x10-14 | | Yes | | 0% | | No | | No | | Yes | | IV | | II | | Moderate | |  |
| Li, 2014 | OSAS | | OSA vs non-OSA | | 5 | | 931  / 3743 | | RR | | 1.376  ( 1.205, 1.572 ) | | 2.6x10-6 | | No | | 10.22% | | No | | Yes | | Yes | | IV | | III | | High | |  |
| Atti, 2019 | Renal sympathetic denervation | | Renal sympathetic denervation  performed  vs not performed | | 6 | | 222/  432 | | RR | | 0.586  ( 0.472, 0.729) | | 1.4x10-6 | | Yes | | 0% | | No | | No | | Yes | | IV | | III | | Moderate | |  |
| Zhuang, 2013 | High BMI (≥25 kg/m2) | | high BMI vs normal BMI | | 8 | | 804/ 2441 | | OR | | 1.558  (1.222, 1.987) | | 4.0x10-4 | | Yes | | 29.37% | | No | | NP | | Yes | | IV | | III | | Moderate | |  |
| Correia 2019 | LA stiffness | | increased stiffness vs decreased stiffness | | 3 | | 340 /  649 | | HR | | 3.551 (1.805 , 6.985 ) | | 2.0x10-4 | | Yes | | 66.84 % | | No | | No | | Yes | | IV | | III | | Critically low | |  |
| Pranata , 2019 | P wave duration | | prolonged p wave duration vs normal duration | | 6 | | 278 /  787 | | OR | | 4.169 ( 2.101, 8.274) | | 4.5x10-5 | | Yes | | 71.38 % | | Yes | | Yes | | No | | IV | | III | | High | |  |
| Chen 2020 | High-power short duration radiofrequency ablation | | high-power short-duration vs low-power long-duration | | 3 | | 454/607 | | RR | | 1.184  (1.071, 1.308) | | 0.001 | | Yes | | 28.89% | | No | | Yes | | Yes | | IV | | IV | | High | |  |
| Vallakati, 2017 | Female sex | | women vs men | | 20 | | 2755/9968 | | OR | | 1.198  (1.043, 1.377]) | | 0.010 | | Yes | | 25.56% | | No | | NP | | Yes | | IV | | IV | | Moderate | |  |
| Ng, 2011 | OSAS | | OSA vs non-OSA | | 6 | | 1157/ 3995 | | RR | | 1.251 (1.082, 1.447 ) | | 0.003 | | Yes | | 48.75 % | | No | | No | | Yes | | IV | | IV | | Critically low | |  |
| Lee , 2021 | CKD | | CKD vs non-CKD | | 8 | | 3267 /23566 | | OR | | 2.991 (1.518 , 5.892 ) | | 0.002 | | Yes | | 87.95 % | | Yes | | Yes | | No | | IV | | IV | | Critically low | |  |
| Njoku 2018 | LAV | | larger LAVI vs smaller LAVI | | 13 | | 84/2693 | | OR | | 1.032  (1.012, 1.052) | | 0.002 | | Yes | | 80.16% | | Yes | | Yes | | Yes | | IV | | IV | | Critically low | |  |
| Tse, 2018 | Inter-atrial block | | Inter-atrial block vs no inter-atrial block | | 3 | | 182/ 265 | | HR | | 2.586  (1.350, 4.955) | | 0.004 | | Yes | | 67.26% | | No | | No | | Yes | | IV | | IV | | Critically low | |  |
| Peng 2020 | | RAAS | | Use or not | | 15 | | NR/4302 | | RR | | 0.83  (0.7, 0.98) | | 0.026 | | Yes | | 68.5 | | No | | NA | | No | | IV | | IV | | Low | |
| Lei 2018 | | steroids | | Use or not | | 5 | | NR/771 | | RR | | 0.67  (0.47, 0.95) | | 0.023 | | Yes | | 0% | | No | | NA | | No | | IV | | IV | | Critically low | |
| Zhang, 2018 | prolonged corrected QT interval | | prolonged corrected QT interval vs control | | 3 | | 366/415 | | HR | | 1.033  ( 0.987, 1.082) | | 0.162 | | Yes | | 86.77% | | Yes | | Yes | | Yes | | NS | | NA | | High | |  |
| McLellan, 2013 | Adenosine | | acute reconnection vs no reconnection | | 6 | | 366/554 | | RR | | 0.913  (0.807,1.033) | | 0.148 | | Yes | | 0% | | No | | No | | No | | NS | | NA | | Critically low | |  |
| Huang 2020 | Low fluoroscopy | | Low-fluoscopy vs Conventional | | 9 | | 291/1430 | | OR | | 1.343  (0.771, 2.340) | | 0.297 | | Yes | | 65.29% | | No | | No | | No | | NS | | NA | | Moderate | |  |
| Zhao , 2016 | serum uric acid | | Increased vs decreased | | 4 | | 932 /1298 | | OR | | 1.370  (0.977, 1.923) | | 0.068 | | Yes | | 99.97% | | No | | Yes | | No | | NS | | NA | | Critically low | |  |
| Pranata , 2020 | serum galectin-3 level | | high vs low | | 3 | | NR/ 180 | | HR | | 1.185  (0.988, 1.422) | | 0.067 | | Yes | | 75.03% | | Yes | | NA | | Yes | | NS | | NA | | Critically low | |  |
| Aldaas 2020 | HFpEF vs HFrEF | | HFpEF vs HFrEF | | 6 | | 436/1283 | | RR | | 1.015  ( 0.765, 1.346) | | 0.917 | | Yes | | 57.41% | | No | | No | | Yes | | NS | | NA | | Moderate | |  |
| Peng, 2018 | statins | | statins vs control | | 10 | | 545 / 1,607 | | OR | | 0.808  (0.592, 1.103) | | 0.1796 | | Yes | | 40.77% | | No | | No | | No | | NS | | NA | | High | |  |
| **Post cardioversion** | | | | | | | | | | | | | | | | | | | | | | | | | | | | | | |  |
| Yo 2014 | High-sensitivity C-reactive protein(hs-CRP) | | high hs-CRP vs low hs-CRP | | 9 | | 347/682 | | OR | | 6.119  (4.055, 9.232) | | 6.1x10-18 | | No | | 14.57% | | Yes | | Yes | | Yes | | IV | | II | | Critically low | |  |
| Dentali 2011 | statins | | statins vs control | | 12 | | 785/1791 | | RR | | 0.777  (0.671, 0.900) | | 7.7x10-4 | | Yes | | 16.44% | | No | | No | | No | | IV | | III | | Critically low | |  |
| Vitali 2019 | CHA2DS2-VASc score | | CHA2DS2-VASc ≥2 vs CHA2DS2-VASc <2 | | 3 | | 89/478 | | OR | | 1.109  (1.049, 1.174) | | 3.0x10-4 | | Yes | | 0% | | No | | NP | | Yes | | IV | | III | | Critically low | |  |

Abbreviations: AF: Atrial Fibrillation; BMI: Body mass index; CA: catheter ablation; CE, class of evidence; CES: class of evidence sensitivity analysis; CHADS VASc: Congestive heart failure, Hypertension, Age>75 years, Diabetes, Stroke, Vascular disease, Age>65, female Sex; CI, confidence interval; CPAP: continuous positive airway pressure; ES, effect size; DC: direct current; ESB, excess significance bias; HF: heart failure; HFpEF: heart failure with preserved ejection fraction; HFrEF: heart failure with reduced ejection fraction; hsCRP: high sensitive C-reactive protein; HR, hazard ratio; i: indexed; I2, heterogeneity; K, number of studies for each factor; LA: left atrium; LAV: left atrial volume; LS, largest study with significant effect; MA, meta-analysis; n, number of cases; N, total number of cohorts per factor; NA, not assessable; NP, not pertinent, because the number of observed studies is less than the expected; NR, not reported; OR, odds ratio; OSAS: obstructive sleep apnea syndrome; PI, prediction interval; RR, risk ratio; SSE, small study effects.; RCT: randomized controlled trial.

Table 2 Predictors for AF recurrence, in meta-analyses of randomized controlled trials.

| **Author, Year** | **predictor** | **Exposed / Unexposed as in included MA** | **K** | **n / N** | **Metric** | **ES (95% CI)** | **p** | **PI include null value** | **I^2^** | **SSE** | **ESB** | **High RoB** | **GLE** | **AMSTAR 2 Quality** |
| --- | --- | --- | --- | --- | --- | --- | --- | --- | --- | --- | --- | --- | --- | --- |
| **Post cardioversion** | | | | | | | | | | | | | | |
| Cao, 2012 | Omega-3 fatty acids | Omega-3 fatty acids vs control | 6 | 455 /  759 | OR | 0.626  (0.348, 1.127 ) | 0.118 | Yes | 66.43% | No | NP | >25 | N/S | Critically low |
| Lafuente , 2015 | Quinidine | Quinidine vs control | 7 | 1158 /  1624 | OR | 0.508  ( 0.401 , 0.401 ) | 0.000 | No | 0% | No | No | >25 | Moderate | High |
| Lafuente , 2015 | Disopyramide | Disopyramide vs control | 2 | 89 /  146 | OR | 0.525  (0.271 , 1.019) | 0.057 | NA | 0% | NA | NP | >25 | N/S | High |
| Lafuente , 2015 | Flecainide | Flecainide vs control | 4 | 276 /  511 | OR | 0.350  (0.200 , 0.615 ) | 0.0000 | No | 34.38 % | No | No | >25 | Moderate | High |
| Lafuente , 2015 | Propafenone | Propafenone | 5 | 652 /  1098 | OR | 0.366  (0.282, 0.476) | 0.027 | Yes | 0% | No | NP | >25 | Moderate | High |
| Lafuente , 2015 | Class I others | Class I others vs control | 3 | 1138 /  1383 | OR | 0.718  (0.422, 1.221) | 0.221 | Yes | 33.02% | No | NP | >25 | N/S | High |
| Lafuente , 2015 | Beta-blockers | Beta-blockers vs control | 2 | 375 /  562 | OR | 0.432  (0.143, 1.300) | 0.135 | NA | 88.60% | NA | No | >25 | Low | High |
| Lafuente , 2015 | Amiodarone | Amiodarone vs control | 6 | 471 /  812 | OR | 0.189  (0.141, 0.253) | 0.0000 | No | 0% | No | No | >25 | Moderate | High |
| Lafuente , 2015 | Azimilide | Azimilide vs control | 4 | 1260/  1602 | OR | 0.702(0.549, 0.897) | 0.0046 | Yes | 0% | No | No | >25 | Moderate | High |
| Lafuente , 2015 | Dofetilide | Dofetilide vs control | 3 | 811 /  1183 | OR | 0.338(0.193, 0.593) | 0.0002 | Yes | 74.85% | Yes | NP | >25 | Moderate | High |
| Lafuente , 2015 | Dronedarone | Dronedarone vs control | 2 | 1001 /  1443 | OR | 0.585(0.459, 0.745) | 0.0000 | NA | 0% | NA | NP | >25 | Moderate | High |
| Lafuente , 2015 | Sotalol | Sotalol vs control | 13 | 2189 /  2758 | OR | 0.512(0.404, 0.647) | 0.0000 | No | 42.71 % | No | Yes | >25 | Moderate | High |
| Lafuente , 2015 | Antiarrythmics | Use or not | 51 | 9420/  13122 | OR | 0.433(0.374, 0.500) | 0.0000 | No | 63.43% | Yes | No | >25 | Moderate | High |
| Lafuente , 2015 | Class Ia | Class Ia vs control | 8 | 1214/  1716 | OR | 0.507(0.404 , 0.637) | 0.0000 | No | 0% | No | No | >25 | Moderate | High |
| Lafuente , 2015 | Class Ib | Class Ib vs control | 2 | 1092/  1321 | OR | 0.845(0.631, 1.132) | 0.259 | NA | 0% | NA | NP | >25 | N/S | High |
| Lafuente , 2015 | Class Ic | Class Ic vs control | 11 | 1010 /  1765 | OR | 0.357 (0.290, 0.441) | 0.0000 | No | 0% | No | NP | >25 | Moderate | High |
| Lafuente , 2015 | Class I | Class I vs control | 21 | 3316 /  4802 | OR | 0.441(0.362, 0.538) | 0.0000 | No | 40.61% | Yes | Yes | >25 | Moderate | High |
| Lafuente , 2015 | Class III | Class III vs control | 27 | 5693 /  8004 | OR | 0.428 ( 0.428 , 0.529 ) | 0.0000 | Yes | 72.12 % | Yes | No | >25 | Moderate | High |
| Lafuente , 2015 | Disopyramide | Disopyramide vs other Class I antiarrythmics | 2 | 53 /  113 | OR | 0.755(0.358, 1.590) | 0.459 | NA | 0% | NA | NP | >25 | N/S | High |
| Lafuente , 2015 | Amiodarone | Amiodarone vs Class I drugs | 5 | 371 /  643 | OR | 0.356(0.239, 0.532) | 0.0000 | Yes | 31.41% | No | NP | >25 | Moderate | High |
| Lafuente , 2015 | Class III | Class III drugs vs Class I drugs | 13 | 1772/  2875 | OR | 0.811(0.588, 1.119) | 0.201 | Yes | 69.68% | No | NP | >25 | N/S | High |
| Schneider 2010 | RAAS | Use or not | 8 | 990/3044 | RR | 0.53  (0.33, 0.85) | 0.009 | Yes | 0% | No | Yes | N/R | Low | Critically Low |
| **Post ablation** | | | | | | | | | | | | | | |
| Chen 2016 | Short-term Antiarrythmics (early recurrence of AF) | Use or not | 6 | 1129 /  2667 | RR | 0.679 ( 0.526 , 0.877 ) | 0.003 | Yes | 58.38 % | Yes | Yes | >25 | Moderate | High |
| Chen 2016 | Short-term Antiarrythmics (late recurrence of AF) | Use or not | 6 | 849 /  2655 | RR | 0.917 ( 0.917 , 1.023 ) | 0.120 | Yes | 0% | No | NP | >25 | N/S | High |

Abbreviations: CE, class of evidence; CI, confidence interval; ES, effect size; ESB, excess significance bias; GLE: GRADE level of evidence; GRADE: GRADE, Grading of Recommendations Assessment, Development and Evaluation; I2, heterogeneity; K, number of studies for each factor; LS, largest study with significant effect; n, number of cases; N, total number of cohort per factor; NA, not assessable; NP, not pertinent, because the number of observed studies is less than the expected; NR, not reported; OR, odds ratio; PI, prediction interval; RoB, risk of bias; RR, risk ratio; SSE, small study effects.
