# Supplementary material for: Predictors of recurrence after catheter ablation and electrical cardioversion of atrial fibrillation: an umbrella review of meta-analyses
Source: Europace. 2022 Aug 29;25(1):40–8. doi: 10.1093/europace/euac143 (PMC10103559; doi:10.1093/europace/euac143)
Supplement: euac143_Supplementary_Data [file euac143_supplementary_data.zip › Supplement recurrence EP-revised.docx]

**Supplementary content**

Contents

[**eAppendix 1** PRISMA 2020 and MOOSE Checklist 2](#_Toc105320035)

[PRISMA 2020 Checklist 2](#_Toc105320036)

[PRISMA 2020 for Abstracts Checklist 4](#_Toc105320037)

[MOOSE Checklist for Meta-analyses of Observational Studies 6](#_Toc105320038)

[**Appendix 2** Search Strategy ***** 9](#_Toc105320039)

[**Appendix 3** List of studies excluded after full-text evaluation with reasoning 10](#_Toc105320040)

[**Appendix 4** Criteria for evaluation of the credibility of evidence 12](#_Toc105320041)

[**Appendix 5** List of studies included in the umbrella meta-analysis with baseline characteristics 13](#_Toc105320042)

[References 18](#_Toc105320043)

# **eAppendix 1** PRISMA 2020 and MOOSE Checklist

## PRISMA 2020 Checklist

| **Section and Topic** | **Item #** | **Checklist item** | **Location where item is reported (page)** |
| --- | --- | --- | --- |
| **TITLE** | | |  |
| Title | 1 | Identify the report as a systematic review. | 1 |
| **ABSTRACT** | | |  |
| Abstract | 2 | See the PRISMA 2020 for Abstracts checklist. | 2, main text  4,5 supplement |
| **INTRODUCTION** | | |  |
| Rationale | 3 | Describe the rationale for the review in the context of existing knowledge. | 3,4 |
| Objectives | 4 | Provide an explicit statement of the objective(s) or question(s) the review addresses. | 4 |
| **METHODS** | | |  |
| Eligibility criteria | 5 | Specify the inclusion and exclusion criteria for the review and how studies were grouped for the syntheses. | 4,5 |
| Information sources | 6 | Specify all databases, registers, websites, organisations, reference lists and other sources searched or consulted to identify studies. Specify the date when each source was last searched or consulted. | 4, 5 Appendix 2 |
| Search strategy | 7 | Present the full search strategies for all databases, registers and websites, including any filters and limits used. | 4, 5 Appendix 2 |
| Selection process | 8 | Specify the methods used to decide whether a study met the inclusion criteria of the review, including how many reviewers screened each record and each report retrieved, whether they worked independently, and if applicable, details of automation tools used in the process. | 4,5 |
| Data collection process | 9 | Specify the methods used to collect data from reports, including how many reviewers collected data from each report, whether they worked independently, any processes for obtaining or confirming data from study investigators, and if applicable, details of automation tools used in the process. | 5,6 |
| Data items | 10a | List and define all outcomes for which data were sought. Specify whether all results that were compatible with each outcome domain in each study were sought (e.g. for all measures, time points, analyses), and if not, the methods used to decide which results to collect. | 5,6 |
|  | 10b | List and define all other variables for which data were sought (e.g. participant and intervention characteristics, funding sources). Describe any assumptions made about any missing or unclear information. | 5,6 |
| Study risk of bias assessment | 11 | Specify the methods used to assess risk of bias in the included studies, including details of the tool(s) used, how many reviewers assessed each study and whether they worked independently, and if applicable, details of automation tools used in the process. | 6 |
| Effect measures | 12 | Specify for each outcome the effect measure(s) (e.g. risk ratio, mean difference) used in the synthesis or presentation of results. | 6,7 |
| Synthesis methods | 13a | Describe the processes used to decide which studies were eligible for each synthesis (e.g. tabulating the study intervention characteristics and comparing against the planned groups for each synthesis (item #5)). | 6,7 |
|  | 13b | Describe any methods required to prepare the data for presentation or synthesis, such as handling of missing summary statistics, or data conversions. | 6,7 |
|  | 13c | Describe any methods used to tabulate or visually display results of individual studies and syntheses. | 6,7 |
|  | 13d | Describe any methods used to synthesize results and provide a rationale for the choice(s). If meta-analysis was performed, describe the model(s), method(s) to identify the presence and extent of statistical heterogeneity, and software package(s) used. | 6,7, Appendix 2 |
|  | 13e | Describe any methods used to explore possible causes of heterogeneity among study results (e.g. subgroup analysis, meta-regression). | 6,7 |
|  | 13f | Describe any sensitivity analyses conducted to assess robustness of the synthesized results. | Table 1 |
| Reporting bias assessment | 14 | Describe any methods used to assess risk of bias due to missing results in a synthesis (arising from reporting biases). | 6,7 |
| Certainty assessment | 15 | Describe any methods used to assess certainty (or confidence) in the body of evidence for an outcome. | 7 |
| **RESULTS** | | |  |
| Study selection | 16a | Describe the results of the search and selection process, from the number of records identified in the search to the number of studies included in the review, ideally using a flow diagram. | 8, Figure 1 |
|  | 16b | Cite studies that might appear to meet the inclusion criteria, but which were excluded, and explain why they were excluded. | Appendix 4 |
| Study characteristics | 17 | Cite each included study and present its characteristics. | Appendix 5 |
| Risk of bias in studies | 18 | Present assessments of risk of bias for each included study. | Appendix 5 |
| Results of individual studies | 19 | For all outcomes, present, for each study: (a) summary statistics for each group (where appropriate) and (b) an effect estimate and its precision (e.g. confidence/credible interval), ideally using structured tables or plots. | 8-10, Table 1, 2 |
| Results of syntheses | 20a | For each synthesis, briefly summarise the characteristics and risk of bias among contributing studies. | Table 1, 2 |
|  | 20b | Present results of all statistical syntheses conducted. If meta-analysis was done, present for each the summary estimate and its precision (e.g. confidence/credible interval) and measures of statistical heterogeneity. If comparing groups, describe the direction of the effect. | 8-10, Table 1, 2 |
|  | 20c | Present results of all investigations of possible causes of heterogeneity among study results. | 8-10, Table 1, 2 |
|  | 20d | Present results of all sensitivity analyses conducted to assess the robustness of the synthesized results. | 8-10, Table 1 |
| Reporting biases | 21 | Present assessments of risk of bias due to missing results (arising from reporting biases) for each synthesis assessed. | 8-10, Table 1, 2 |
| Certainty of evidence | 22 | Present assessments of certainty (or confidence) in the body of evidence for each outcome assessed. | 8-10, Table 1, 2 |
| **DISCUSSION** | | |  |
| Discussion | 23a | Provide a general interpretation of the results in the context of other evidence. | 10-12 |
|  | 23b | Discuss any limitations of the evidence included in the review. | 10-13 |
|  | 23c | Discuss any limitations of the review processes used. | 13 |
|  | 23d | Discuss implications of the results for practice, policy, and future research. | 13 |
| **OTHER INFORMATION** | | |  |
| Registration and protocol | 24a | Provide registration information for the review, including register name and registration number, or state that the review was not registered. | 2 |
|  | 24b | Indicate where the review protocol can be accessed, or state that a protocol was not prepared. | 2 |
|  | 24c | Describe and explain any amendments to information provided at registration or in the protocol. | N/A |
| Support | 25 | Describe sources of financial or non-financial support for the review, and the role of the funders or sponsors in the review. | 13 |
| Competing interests | 26 | Declare any competing interests of review authors. | 13 |
| Availability of data, code and other materials | 27 | Report which of the following are publicly available and where they can be found: template data collection forms; data extracted from included studies; data used for all analyses; analytic code; any other materials used in the review. | 14 |

## PRISMA 2020 for Abstracts Checklist

| **Section and Topic** | **Item #** | **Checklist item** | **Reported (Yes/No)** |
| --- | --- | --- | --- |
| **TITLE** | | |  |
| Title | 1 | Identify the report as a systematic review. | Yes |
| **BACKGROUND** | | |  |
| Objectives | 2 | Provide an explicit statement of the main objective(s) or question(s) the review addresses. | Yes |
| **METHODS** | | |  |
| Eligibility criteria | 3 | Specify the inclusion and exclusion criteria for the review. | No |
| Information sources | 4 | Specify the information sources (e.g. databases, registers) used to identify studies and the date when each was last searched. | Yes |
| Risk of bias | 5 | Specify the methods used to assess risk of bias in the included studies. | Yes |
| Synthesis of results | 6 | Specify the methods used to present and synthesise results. | Yes |
| **RESULTS** | | |  |
| Included studies | 7 | Give the total number of included studies and participants and summarise relevant characteristics of studies. | Yes |
| Synthesis of results | 8 | Present results for main outcomes, preferably indicating the number of included studies and participants for each. If meta-analysis was done, report the summary estimate and confidence/credible interval. If comparing groups, indicate the direction of the effect (i.e. which group is favoured). | Yes |
| **DISCUSSION** | | |  |
| Limitations of evidence | 9 | Provide a brief summary of the limitations of the evidence included in the review (e.g. study risk of bias, inconsistency and imprecision). | Yes |
| Interpretation | 10 | Provide a general interpretation of the results and important implications. | Yes |
| **OTHER** | | |  |
| Funding | 11 | Specify the primary source of funding for the review. | NA due to the journal requirements |
| Registration | 12 | Provide the register name and registration number. | Yes |

## MOOSE Checklist for Meta-analyses of Observational Studies

| **Reporting Criteria** | **Reported (Yes/No)** | **Reported on Page** |
| --- | --- | --- |
| **Reporting of Background** |  |  |
| Problem definition | Yes | 3,4 |
| Hypothesis statement | Yes | 4 |
| Description of Study Outcome(s) | Yes | 4,5 |
| Type of exposure or intervention used | Yes | 4,5 |
| Type of study design used | Yes | 4,5 |
| Study population | Yes | 4,5 |
| **Reporting of Search Strategy** |  |  |
| Qualifications of searchers (eg, librarians  and investigators) | NA |  |
| Search strategy, including time period  included in the synthesis and keywords | Yes | 4,5 and Appendix 2 |
| Effort to include all available studies,  including contact with authors | Yes | 5 |
| Databases and registries searched | Yes | 4,5 |
| Search software used, name and  version, including special features used  (eg, explosion) | NA |  |
| Use of hand searching (eg, reference  lists of obtained articles) | Yes | 5 |
| List of citations located and those  excluded, including justification | Yes | Figure 1, Appendix 4, 5 |
| Method for addressing articles  published in languages other than  English | We placed restrictions on English |  |
| Method of handling abstracts and  unpublished studies | NA |  |
| Description of any contact with authors | NA |  |
| **Reporting of Methods** |  |  |
| Description of relevance or  appropriateness of studies assembled for  assessing the hypothesis to be tested | Yes | 5,6 |
| Rationale for the selection and coding of  data (eg, sound clinical principles or  convenience) | Yes | 5,6 |
| Documentation of how data were  classified and coded (eg, multiple raters,  blinding, and interrater reliability) | Yes | 6,7 |
| Assessment of confounding (eg,  comparability of cases and controls in  studies where appropriate | Yes | 6,7 |
| Assessment of study quality, including  blinding of quality assessors;  stratification or regression on possible  predictors of study results YES 5 | Yes | 6,7 |
| Assessment of heterogeneity | Yes | 6,7 |
| Description of statistical methods (eg,  complete description of fixed or random  effects models, justification of whether  the chosen models account for predictors  of study results, dose-response models,  or cumulative meta-analysis) in sufficient  detail to be replicated | Yes | 6,7 |
| Provision of appropriate tables and  graphics | Yes | Figure 1, Table 1, 2 |
| **Reporting of Results** |  |  |
| Table giving descriptive information for  each study included | Yes | Appendix 5 |
| Results of sensitivity testing (eg,  subgroup analysis) | Yes | 8-9, Table 1, 2 |
| Indication of statistical uncertainty of  findings | Yes | 8-9, Table 1,2 |
| **Reporting of Discussion** |  |  |
| Quantitative assessment of bias (eg,  publication bias) | Yes | Table 1, 2 |
| Justification for exclusion (eg, exclusion  of non–English-language citations) | Yes | Appendix 2, 4 |
| Assessment of quality of included studies | Yes | Table 1, 2 |
| **Reporting of Conclusions** |  |  |
| Consideration of alternative explanations  for observed results | Yes | 13,14 |
| Generalization of the conclusions (ie,  appropriate for the data presented and  within the domain of the literature review) | Yes | 13,14 |
| Guidelines for future research | Yes | 13,14 |
| Disclosure of funding source | Yes | 14 |

# **Appendix 2** Search Strategy *****

**Pubmed**

(auricular fibrillation OR atrial fibrillation OR AF OR AFib) AND (“meta-analysis as topic”[MeSH:noexp] OR Meta-Analysis[ptyp] OR metaanaly*[tiab] OR meta-analy*[tiab])

**Web of science**

|  |  | (TS=(atrial fibrillation AND TS= (meta-analysis OR metaanaly* OR meta-analy*) *AND* **LANGUAGE:** (English) *AND* **DOCUMENT TYPES:** (Article)  **Cochrane review** |
| --- | --- | --- |

**Keyword:**

Atrial fibrillation

**Embase (Cochrane central database of triasls (373)**

(auricular fibrillation OR atrial fibrillation) AND (meta-analysis OR metaanaly* OR meta-analy*)

*While our search had no language restriction; nevertheless, only meta-analyses published in English were finally included due to lack of translation resources for other languages.

# **Appendix 3** List of studies excluded after full-text evaluation with reasoning

| **Author, Year** | **Reason for exclusion** |
| --- | --- |
| Chaugai, 2016(1) | Another outcome |
| Zhang, 2016(2) | Different estimate measures |
| Disertori, 2012(3) | Another outcome |
| Jibrini, 2008(4) | Another meta-analysis with larger number of studies with same risk or protective factor |
| Zhang, 2010(5) | Another outcome |
| Bhuriya, 2011(6) | Another outcome |
| Samokhvalov, 2010(7) | Another outcome |
| Kodama, 2011(8) | Another outcome |
| Tang, 2011(9) | Different estimate measures |
| Zografos, 2014(10) | Different estimate measures |
| Xu, 2017(11) | Different estimate measures |
| Loke, 2009(12) | Systematic review (no meta-analysis) |
| Kim, 2010(13) | Another outcome |
| Sharma, 2013(14) | Another meta-analysis with larger number of studies with same risk or protective factor |
| Mak, 2009(15) | Another outcome |
| Howard, 2010(16) | Systematic review (no meta-analysis) |
| Deftereos, 2019(17) | Systematic review (no meta-analysis) |
| Leong, 2016(18) | Another meta-analysis with larger number of studies with same risk or protective factor |
| Tanboga,2016(19) | Another outcome |
| Liu, 2016(20) | Another outcome |
| Nomani, 2020(21) | Systematic review (no meta-analysis) |
| Kontogiorgis, 2016(22) | Systematic review (no meta-analysis) |
| Chokesuwattanaskul, 2020(23) | Including cross-sectional studies |
| Madrid, 2004(24) | Another meta-analysis with larger number of studies with same risk or protective factor |
| Makkar, 2009(25) | Another outcome |
| Dagres, 2014(26) | Different estimate measures |
| De Vecchis, 2018(27) | Another meta-analysis with larger number of studies with same risk or protective factor |
| Yang, 2014(28) | Another meta-analysis with larger number of studies with same risk or protective factor |
| Santangeli, 2010(29) | Another outcome |
| Fauchier, 2008(30) | Another meta-analysis with larger number of studies with same risk or protective factor |
| Liu, 2008(31) | Another meta-analysis with larger number of studies with same risk or protective factor |
| Rahimi, 2011(32) | Another meta-analysis with larger number of studies with same risk or protective factor |
| Fang, 2012(33) | Another meta-analysis with larger number of studies with same risk or protective factor |
| Fauchier, 2013(34) | Another meta-analysis with larger number of studies with same risk or protective factor |
| Zhou, 2013(35) | Another meta-analysis with larger number of studies with same risk or protective factor |
| Bhardwaj, 2010(36) | Another meta-analysis with larger number of studies with same risk or protective factor |
| Corrado, 2008(37) | Another meta-analysis with larger number of studies with same risk or protective factor |
| Loffredo, 2012(38) | Another meta-analysis with larger number of studies for the same risk or protective factor of AF |
| Yan, 2014(39) | Another meta-analysis with larger number of studies for the same risk or protective factor of AF |
| He, 2013(40) | Another meta-analysis with larger number of studies for the same risk or protective factor of AF |
| Jiang, 2018(41) | Another meta-analysis with larger number of studies for the same risk or protective factor of AF |
| Bajraktari, 2020(42) | Different estimate measures |
| Nielsen, 2020(43) | Different estimate measures |
| Jin, 2018(44) | Different estimate measures |
| Zhuang, 2012(45) | Different estimate measures |
| Froehlich, 2019(46) | Another outcome |
| D'Ascenzo, 2013(47) | Different estimate measures |
| Jiang, 2013(48) | Different estimate measures |
| Liu, 2008(49) | Different estimate measures |
| Labarca, 2020(50) | Another outcome |
| Jiang, 2017(51) | Different estimate measures |
| Wang, 2017(52) | Another meta-analysis with larger number of studies for the same risk or protective factor of AF |
| Noubiap, 2019(53) | Another outcome |
| Weymann, 2017(54) | Different estimate measures |
| Bai, 2018(55) | Different estimate measures |
| Guijian, 2013(56) | Another meta-analysis with larger number of studies for the same risk or protective factor of AF |
| Li, 2016(57) | Different estimate measures |
| Sepehri Shamloo, 2019(58) | Different estimate measures |
| Burgess, 2006(59) | Another outcome |
| Mujer, 2020(60) | Another meta-analysis with larger number of studies for the same risk or protective factor of AF |
| Wilson, 2020(61) | Another meta-analysis with larger number of studies for the same risk or protective factor of AF |
| Salih, 2020(62) | Another outcome |
| Kewcharoen, 2020(63) | Another meta-analysis with larger number of studies for the same risk or protective factor of AF |
| Kewcharoen, 2020(64) | Another outcome |
| Shi, 2020(65) | Another outcome |

# **Appendix 4** Criteria for evaluation of the credibility of evidence

eTable 1. Criteria for evaluation of the credibility of the evidence of observational studies

| **Classification** | **Criteria** |
| --- | --- |
| Convincing evidence (Class I) | 1. More than 1000 cases 2. Significant summary associations (p<1x10^-6^) per random-effects calculations 3. No evidence of small-study effects 4. No evidence of excess of significance bias 5. Prediction intervals not including the null value 6. Largest study nominally significant (p<0.05) 7. No large heterogeneity (i.e., *I^2^*< 50%) |
| Highly Suggestive evidence (Class II) | 1. More than 1000 cases 2. Significant summary associations (p<1x10^-6^) per random-effects calculation 3. Largest study nominally significant (p<0.05) |
| Suggestive Evidence (Class III) | 1. More than 1000 cases 2. Significant summary associations (p<1x10^-3^) per random-effects calculations |
| Weak evidence (Class IV) | 1. All other associations with p≤0.05 |
| Non-significant associations (NS) | 1. All associations with p >0.05 |

eTable 2. Criteria for evaluation of the credibility of the evidence of RCTs

| **Grade level of evidence** | **Number of downgrades** |
| --- | --- |
| High | 0 downgrades |
| Moderate | 1 -2 downgrades |
| Low | 3-4 downgrades |
| Very Low | 5-6 downgrades |

Note: GRADE: Grading of Recommendations Assessment,Development and Evaluation.

# **Appendix 5** List of studies included in the umbrella meta-analysis with baseline characteristics

| **Study** | **Type of studies included** | **Risk or protective factors of recurrence after catheter ablation or DC-konversion of AF** | **Exposures** | **Non-exposures (comparator)** | **No of included studies estimates** | **Sample size** | **Population (s)** | **AMSTAR 2** |
| --- | --- | --- | --- | --- | --- | --- | --- | --- |
| Atti, 2019(66) | Prospective cohort, RCTs | Renal sympathetic denervation | Renal sympathetic denervation | Renal sympathetic denervation not performed | 6 | 432 | Hypertensive  patients with AF eligible for CA | Moderate quality |
| Vallakati, 2015(67) | Retrospective cohorts | Gender | Women | Men | 20 | 9968 | Adult AF patients eligible for CA | Moderate quality |
| Li, 2014(68) | Retrospective cohort, Case-controls | OSAS | OSAS | Non-OSAS | 5 | 3743 | Patients with AF with eligible for CA | High quality |
| Ng, 2011(69) | Prospective cohorts | OSAS | OSAS | Non-OSAS | 6 | 3995 | Patients with AF with eligible for CA | Critical Low |
| Zhuang, 2013(70) | Prospective cohort, Retrospective cohort | BMI | High BMI (≥25 kg/m2) | Normal BMI | 8 | 2441 | AF patients undergoing  CA | Moderate quality |
| Yo, 2014(71) | Prospective cohorts, Retrospective cohorts | hsCRP | High hs-CRP | Low hs-CRP | 9 | 682 | Patients with AF eligible for DC-conversion | Critially low quality |
| Peng, 2018(72) | RCTs, Retrospective cohorts | Statins | Statin use | No use | 10 | 1607 | Patients with AF and eligible for CA | High quality |
| Dentali, 2011(73) | RCTs, Retrospective cohorts, Prospective cohorts | Statin | Statin use | No use | 12 | 1791 | Patients with AF eligible for DC-conversion | Critially low quality |
| Cao, 2012(74) | RCTs | Omega-3 fatty acids | Omega-3 fatty acids use | No use | 6 | 759 | Patients with AF eligible for DC-conversion | Critially low quality |
| Schneider, 2010(75) | RCTs | RAAS blockers | Use of RAAS blockers | No use | 8 | 3044 | Patients with AF eligible for DC-conversion | Critially low quality |
| Chew, 2020(76) | Retrospective cohorts | AF Diagnosis-to-Ablation Time (DAT) | DAT ≤ 1 year | DAT > 1 year | 6 | 4950 | Patients with AF eligible for CA | High quality |
| Pranata, 2019(77) | Prospective cohorts | p-wave duration | Prolonged p-wave duration | Normal p-wave duration | 6 | 787 | Patients with AF eligible for CA | High quality |
| Peng, 2020(78) | RCTs, prospective and retrospective cohorts | RAAS blockers | Use of RAAS blockers | No use | 15 | 4302 | Patients with AF eligible for CA | Low quality |
| Shukla, 2015(79) | Prospective cohorts | CPAP in patients with OSAS | CPAP use | No CPAP use | 7 | 1087 | Patients with OSAS and AF eligible for CA | Moderate quality |
| Zhao, 2016(80) | Prospective cohorts, Retrospective cohorts | serum uric acid | Increased levels of serum uric acid | Decreased levels of serum uric acid | 4 | 1298 | Patients with AF eligible for CA | Critially low quality |
| Pranata, 2020(81) | Prospective cohorts | Serum galectin-3 level | High serum galectin-3 levels | Low serum galectin-3 levels | 4 | 340 | Patients with AF eligible for CA | Critially low quality |
| Correia, 2019(82) | Prospective cohorts | LA stiffness | LA stiffness | No LA stiffness | 3 | 922 | Patients with AF eligible for CA | Critially low quality |
| Njoku, 2018(83) | Prospective cohorts, Retrospective cohorts | LAV | Larger LAV/i | Smaller LAV/i | 13 | 2693 | Patients with AF eligible for CA | Critially low quality |
| Tse, 2018(84) | Prospective cohorts, Retrospective cohorts | Inter-atrial block | Inter-atrial block | No Inter-atrial block | 3 | 265 | Patients with AF eligible for DC | Critially low quality |
| Lei, 2018(85) | RCTs and cohorts | Steroids | Steroid use | No use | 5 | 771 | Patients with AF undergoing CA | Critically low quality |
| Zhang, 2018(86) | Prospective cohorts, Retrospective cohorts | QT interval  corrected QT interval | Prolonged corrected QT interval | Normal QT-interval | 3 | 415 | Patients with AF | High quality |
| McLellan, 2013(87) | Prospective cohorts, Retrospective cohorts | Adenosine | Positive adenosine provocation test | Negative provocation test | 6 | 554 | Patients with AF undergoing CA | Critially low quality |
| Chen, 2016(88) | RCTs | Antiarrhythmic drugs | Antiarrhythmic drugs use | No use | 6 | 2667 | Patients with AF undergoing CA | High quality |
| Vitali, 2019(89) | Prospective cohorts | CHA2DS2-VASc | CHA2DS2-VASc ≥2 | CHA2DS2-VASc <2 | 3 | 478 | Patients with AF eligible for DC-conversion | Critially low quality |
| Lafuente-Lafuente, 2015(90) | RCTs | Antiarrhythmic drugs | Use of antiarrhythmic drugs | No use of antiarrhythmic drugs | 7 | 13122 | Patients with AF eligible for DC-conversion | High quality |
| Huang, 2020(91) | Prospective cohorts, Retrospective cohorts | Low fluoroscopy | Low-fluoscopy approach | Conventional approach | 9 | 1430 | Patients with AF undergoing CA | Moderate quality |
| Aldaas, 2021(92) | Retrospective cohorts | HF | HFrEF | HFpEF | 6 | 1283 | Patients with HF eligible for CA of AF | Moderate quality |
| Liu, 2020(93) | Prospective cohort | Burst pacing post CA | AF noninducibility | AF inducibility | 14 | 2628 | Patients with AF undergoing CA | Critially low quality |
| Zhuo, 2020(94) | Prospective and retrospective cohorts | Depression | Depression | No depression | 7 | 985 | Patients with AF eligible for CA | Critially low quality |
| Lin, 2014(95) | Prospective and retrospective cohorts | hypertension | hypertension | No hypertension | 17 | 11430 | Patients with AF undergoing CA | Low quality |
| Lin, 2014(95) | Prospective and retrospective cohorts | Metabolic syndrome | Metabolic syndrome | No metabolic syndrome | 5 | 3320 | Patients with AF undergoing CA | Low quality |
| Lee, 2021(96) | Prospective and retrospective cohorts | Chronic kidney disease | Chronic kidney disease | Normal kidney function | 7 | 23566 | Patients with AF eligible for CA | Critically low quality |

AF: Atrial Fibrillation; BMI: Body mass index; CA: catheter ablation; CHADS VASc: Congestive heart failure, Hypertension, Age>75 years, Diabetes, Stroke, Vascular disease, Age>65, female Sex; CPAP: continuous positive airway pressure; DC: direct current; HF: heart failure; HFpEF: heart failure with preserved ejection fraction; HFrEF: heart failure with reduced ejection fraction; hsCRP: high sensitive C-reactive protein; i: indexed; LA: left atrium; LAV: left atrial volume; OSAS: obstructive sleep apnea syndrome; RCT: randomized controlled trial;

# References

1. Chaugai S, Meng WY, Ali Sepehry A. Effects of RAAS Blockers on Atrial Fibrillation Prophylaxis: An Updated Systematic Review and Meta-Analysis of Randomized Controlled Trials. J Cardiovasc Pharmacol Ther 2016;21:388-404.

2. Zhang CH, Huang DS, Shen D, Zhang LW, Ma YJ, Wang YM, et al. Association Between Serum Uric Acid Levels and Atrial Fibrillation Risk. Cell Physiol Biochem 2016;38:1589-95.

3. Disertori M, Quintarelli S. Renin-Angiotensin System and AtrialFibrillation:Understanding the Connection. J Atr Fibrillation 2011;4:398.

4. Jibrini MB, Molnar J, Arora RR. Prevention of atrial fibrillation by way of abrogation of the renin-angiotensin system: a systematic review and meta-analysis. Am J Ther 2008;15:36-43.

5. Zhang Y, Zhang P, Mu Y, Gao M, Wang JR, Wang Y, et al. The role of renin-angiotensin system blockade therapy in the prevention of atrial fibrillation: a meta-analysis of randomized controlled trials. Clin Pharmacol Ther 2010;88:521-31.

6. Bhuriya R, Singh M, Molnar J, Arora R, Khosla S. Bisphosphonate use in women and the risk of atrial fibrillation: a systematic review and meta-analysis. Int J Cardiol 2010;142:213-7.

7. Samokhvalov AV, Irving HM, Rehm J. Alcohol consumption as a risk factor for atrial fibrillation: a systematic review and meta-analysis. Eur J Cardiovasc Prev Rehabil 2010;17:706-12.

8. Kodama S, Saito K, Tanaka S, Horikawa C, Saito A, Heianza Y, et al. Alcohol consumption and risk of atrial fibrillation: a meta-analysis. J Am Coll Cardiol 2011;57:427-36.

9. Tang Y, Yang H, Qiu J. Relationship between brain natriuretic peptide and recurrence of atrial fibrillation after successful electrical cardioversion: a meta-analysis. J Int Med Res 2011;39:1618-24.

10. Zografos T, Maniotis C, Katsivas A, Katritsis D. Relationship between brain natriuretic peptides and recurrence of atrial fibrillation after successful direct current cardioversion: a meta-analysis. Pacing Clin Electrophysiol 2014;37:1530-7.

11. Xu X, Tang Y. Relationship between Brain Natriuretic Peptide and Recurrence of Atrial Fibrillation after Successful Electrical Cardioversion: an Updated Meta-Analysis. Braz J Cardiovasc Surg 2017;32:530-5.

12. Loke YK, Jeevanantham V, Singh S. Bisphosphonates and atrial fibrillation: systematic review and meta-analysis. Drug Saf 2009;32:219-28.

13. Kim SY, Kim MJ, Cadarette SM, Solomon DH. Bisphosphonates and risk of atrial fibrillation: a meta-analysis. Arthritis Res Ther 2010;12:R30.

14. Sharma A, Chatterjee S, Arbab-Zadeh A, Goyal S, Lichstein E, Ghosh J, et al. Risk of serious atrial fibrillation and stroke with use of bisphosphonates: evidence from a meta-analysis. Chest. 2013;144:1311-22.

15. Mak A, Cheung MWL, Ho RC-M, Cheak AA-C, Lau CS. Bisphosphonates and atrial fibrillation: Bayesian meta-analyses of randomized controlled trials and observational studies. BMC Musculoskelet Disord 2009;10:113.

16. Howard PA, Barnes BJ, Vacek JL, Chen W, Lai S-M. Impact of Bisphosphonates on the Risk of Atrial Fibrillation. Am J Cardiovascul Drugs 2010;10:359-67.

17. Deftereos SG, Vrachatis DA, Angelidis C, Vrettou AR, Sarri EK, Giotaki SG, et al. The Role of Colchicine in Treating Postoperative and Post-catheter Ablation Atrial Fibrillation. Clin Ther 2019;41:21-9.

18. Leong DP, Caron F, Hillis C, Duan A, Healey JS, Fraser G, et al. The risk of atrial fibrillation with ibrutinib use: a systematic review and meta-analysis. Blood 2016;128:138-40.

19. Tanboğa İH, Topçu S, Aksakal E, Gulcu O, Aksakal E, Aksu U, et al. The Risk of Atrial Fibrillation With Ivabradine Treatment: A Meta-analysis With Trial Sequential Analysis of More Than 40000 Patients. Clin Cardiol 2016;39:615-20.

20. Liu T, Korantzopoulos P, Shao Q, Zhang Z, Letsas KP, Li G. Mineralocorticoid receptor antagonists and atrial fibrillation: a meta-analysis. Europace 2016;18:672-8.

21. Nomani H, Saei S, Johnston TP, Sahebkar A, Mohammadpour AH. The Efficacy of Anti-inflammatory Agents in the Prevention of Atrial Fibrillation Recurrences. Curr Med Chem 2021;28:137-51.

22. Kontogiorgis C, Valikeserlis I, Hadjipavlou-Litina D, Nena E, Constantinidis TC. Use of Non-Selective Non-Steroidal Anti-Inflammatory Drugs in Relation to Cardiovascular Events. A Systematic Pharmacoepidemiological Review. Curr Vasc Pharmacol 2016;14:502-13.

23. Chokesuwattanaskul R, Thongprayoon C, Pachariyanon P, Sharma K, Ungprasert P, Bathini T, et al. Erectile dysfunction and atrial fibrillation: A systematic review and meta-analysis. Int J Urol 2018;25:752-7.

24. Madrid AH, Peng J, Zamora J, Marín I, Bernal E, Escobar C, et al. The role of angiotensin receptor blockers and/or angiotensin converting enzyme inhibitors in the prevention of atrial fibrillation in patients with cardiovascular diseases: meta-analysis of randomized controlled clinical trials. Pacing Clin Electrophysiol 2004;27:1405-10.

25. Makkar KM, Sanoski CA, Spinler SA. Role of angiotensin-converting enzyme inhibitors, angiotensin II receptor blockers, and aldosterone antagonists in the prevention of atrial and ventricular arrhythmias. Pharmacotherapy 2009;29:31-48.

26. Dagres N, Varounis C, Gaspar T, Piorkowski C, Eitel C, Iliodromitis EK, et al. Catheter ablation for atrial fibrillation in patients with left ventricular systolic dysfunction. A systematic review and meta-analysis. J Card Fail 2011;17:964-70.

27. De Vecchis R, Ariano C, Giasi A, Cioppa C. Antiarrhythmic effects of ranolazine used both alone for prevention of atrial fibrillation and as an add-on to intravenous amiodarone for its pharmacological cardioversion: a meta-analysis. Minerva Cardioangiol 2018;66:349-59.

28. Yang Q, Qi X, Li Y. The preventive effect of atorvastatin on atrial fibrillation: a meta-analysis of randomized controlled trials. BMC Cardiovasc Disord 2014;14:99.

29. Santangeli P, Ferrante G, Pelargonio G, Dello Russo A, Casella M, Bartoletti S, et al. Usefulness of statins in preventing atrial fibrillation in patients with permanent pacemaker: a systematic review. Europace 2010;12:649-54.

30. Fauchier L, Pierre B, de Labriolle A, Grimard C, Zannad N, Babuty D. Antiarrhythmic effect of statin therapy and atrial fibrillation a meta-analysis of randomized controlled trials. J Am Coll Cardiol 2008;51:828-35.

31. Liu T, Li L, Korantzopoulos P, Liu E, Li G. Statin use and development of atrial fibrillation: a systematic review and meta-analysis of randomized clinical trials and observational studies. Int J Cardiol 2008;126:160-70.

32. Rahimi K, Emberson J, McGale P, Majoni W, Merhi A, Asselbergs FW, et al. Effect of statins on atrial fibrillation: collaborative meta-analysis of published and unpublished evidence from randomised controlled trials. BMJ 2011;342:d1250.

33. Fang W-t, Li H-j, Zhang H, Jiang S. The role of statin therapy in the prevention of atrial fibrillation: a meta-analysis of randomized controlled trials. Br J Clin Pharmacol 2012;74:744-56.

34. Fauchier L, Clementy N, Babuty D. Statin therapy and atrial fibrillation: systematic review and updated meta-analysis of published randomized controlled trials. Curr Opin Cardiol 2013;28:7-18.

35. Zhou X, Du JL, Yuan J, Chen YQ. Statin therapy is beneficial for the prevention of atrial fibrillation in patients with coronary artery disease: a meta-analysis. Eur J Pharmacol 2013;707:104-11.

36. Bhardwaj A, Sood NA, Kluger J, Coleman CI. Lack of effect of statins on maintenance of normal sinus rhythm following electrical cardioversion of persistent atrial fibrillation. Int J Clin Pract 2010;64:1116-20.

37. Corrado A, Raviele A. Antiarrhythmic Effect of Statin Therapy and Atrial Fibrillation A Meta-Analysis of Randomized Controlled Trials. J Atr Fibrillation 2008;1:50.

38. Loffredo L, Angelico F, Perri L, Violi F. Upstream therapy with statin and recurrence of atrial fibrillation after electrical cardioversion. Review of the literature and meta-analysis. BMC Cardiovasc Disord 2012;12:107.

39. Yan P, Dong P, Li Z, Cheng J. Statin therapy decreased the recurrence frequency of atrial fibrillation after electrical cardioversion: a meta-analysis. Med Sci Monit 2014;20:2753-8.

40. He Z, Yang L, Tian J, Yang K, Wu J, Yao Y. Efficacy and safety of omega-3 fatty acids for the prevention of atrial fibrillation: a meta-analysis. Can J Cardiol 2013;29:196-203.

41. Jiang Y, Tan HC, Tam WWS, Lim TW, Wang W. A meta-analysis on Omega-3 supplements in preventing recurrence of atrial fibrillation. Oncotarget 2018;9:6586-94.

42. Bajraktari G, Bytyçi I, Henein MY. Left atrial structure and function predictors of recurrent fibrillation after catheter ablation: a systematic review and meta-analysis. Clin Physiol Funct Imaging. 2020;40:1-13.

43. Nielsen AB, Skaarup KG, Lassen MCH, Djernæs K, Hansen ML, Svendsen JH, et al. Usefulness of left atrial speckle tracking echocardiography in predicting recurrence of atrial fibrillation after radiofrequency ablation: a systematic review and meta-analysis. Int J Cardiovasc Imaging 2020;36:1293-309.

44. Jin X, Pan J, Wu H, Xu D. Are left ventricular ejection fraction and left atrial diameter related to atrial fibrillation recurrence after catheter ablation?: A meta-analysis. Medicine (Baltimore). 2018;97:e10822.

45. Zhuang J, Wang Y, Tang K, Li X, Peng W, Liang C, et al. Association between left atrial size and atrial fibrillation recurrence after single circumferential pulmonary vein isolation: a systematic review and meta-analysis of observational studies. Europace 2011;14:638-45.

46. Froehlich L, Meyre P, Aeschbacher S, Blum S, Djokic D, Kuehne M, et al. Left atrial dimension and cardiovascular outcomes in patients with and without atrial fibrillation: a systematic review and meta-analysis. Heart 2019;105:1884-91.

47. D'Ascenzo F, Corleto A, Biondi-Zoccai G, Anselmino M, Ferraris F, di Biase L, et al. Which are the most reliable predictors of recurrence of atrial fibrillation after transcatheter ablation?: a meta-analysis. Int J Cardiol 2013;167:1984-9.

48. Jiang Z, Dai L, Song Z, Li H, Shu M. Association between C-reactive protein and atrial fibrillation recurrence after catheter ablation: a meta-analysis. Clin Cardiol 2013;36:548-54.

49. Liu T, Li L, Korantzopoulos P, Goudevenos JA, Li G. Meta-analysis of association between C-reactive protein and immediate success of electrical cardioversion in persistent atrial fibrillation. Am J Cardiol 2008;101:1749-52.

50. Labarca G, Dreyse J, Drake L, Jorquera J, Barbe F. Efficacy of continuous positive airway pressure (CPAP) in the prevention of cardiovascular events in patients with obstructive sleep apnea: Systematic review and meta-analysis. Sleep Med Rev 2020;52:101312.

51. Jiang H, Wang W, Wang C, Xie X, Hou Y. Association of pre-ablation level of potential blood markers with atrial fibrillation recurrence after catheter ablation: a meta-analysis. Europace 2017;19(3):392-400.

52. Wang YS, Chen GY, Li XH, Zhou X, Li YG. Prolonged P-wave duration is associated with atrial fibrillation recurrence after radiofrequency catheter ablation: A systematic review and meta-analysis. Int J Cardiol 2017;227:355-9.

53. Noubiap JJ, Bigna JJ, Agbor VN, Mbanga C, Ndoadoumgue AL, Nkeck JR, et al. Meta-analysis of Atrial Fibrillation in Patients With Various Cardiomyopathies. Am J Cardiol 2019;124(2):262-9.

54. Weymann A, Sabashnikov A, Ali-Hasan-Al-Saegh S, Popov AF, Jalil Mirhosseini S, Baker WL, et al. Predictive Role of Coagulation, Fibrinolytic, and Endothelial Markers in Patients with Atrial Fibrillation, Stroke, and Thromboembolism: A Meta-Analysis, Meta-Regression, and Systematic Review. Med Sci Monit Basic Res 2017;23:97-140.

55. Bai Y, Guo SD, Liu Y, Ma CS, Lip GYH. Relationship of troponin to incident atrial fibrillation occurrence, recurrence after radiofrequency ablation and prognosis: a systematic review, meta-analysis and meta-regression. Biomarkers 2018;23:512-7.

56. Guijian L, Jinchuan Y, Rongzeng D, Jun Q, Jun W, Wenqing Z. Impact of Body Mass Index on Atrial Fibrillation Recurrence: A Meta-analysis of Observational Studies. Pacing Clin Electrophysiol 2013;36:748-56.

57. Li J, Yang Y, Ng CY, Zhang Z, Liu T, Li G. Association of Plasma Transforming Growth Factor-β1 Levels and the Risk of Atrial Fibrillation: A Meta-Analysis. PLoS One 2016;11:e0155275.

58. Sepehri Shamloo A, Dagres N, Dinov B, Sommer P, Husser-Bollmann D, Bollmann A, et al. Is epicardial fat tissue associated with atrial fibrillation recurrence after ablation? A systematic review and meta-analysis. Int J Cardiol Heart Vasc 2019;22:132-8.

59. Burgess DC, Kilborn MJ, Keech AC. Interventions for prevention of post-operative atrial fibrillation and its complications after cardiac surgery: a meta-analysis. Eur Heart J 2006;27:2846-57.

60. Mujer MT, Al-Abcha A, Saleh Y, Nerusu LA, Boumegouas M, Herzallah K, et al. Effect of combined renal denervation and pulmonary vein isolation in atrial fibrillation recurrence in hypertensive patients: A meta-analysis. Pacing Clin Electrophysiol 2020;43:866-74.

61. Wilson H, Patton D, Moore Z, O'Connor T, Nugent L. Comparison of dronedarone vs. flecainide in the maintenance of sinus rhythm, following electrocardioversion in adults with persistent atrial fibrillation: a systematic review and meta-analysis. Eur Heart J Cardiovasc Pharmacother 2021;7:363-72.

62. Salih M, Darrat Y, Ibrahim AM, Al-Akchar M, Bhattarai M, Koester C, et al. Clinical outcomes of adjunctive posterior wall isolation in persistent atrial fibrillation: A meta-analysis. J Cardiovasc Electrophysiol 2020;31:1394-402.

63. Kewcharoen J, Vutthikraivit W, Rattanawong P, Prasitlumkum N, Akoum NW, Bunch TJ, et al. Renal sympathetic denervation in addition to pulmonary vein isolation reduces the recurrence rate of atrial fibrillation: an updated meta-analysis of randomized control trials. J Interv Card Electrophysiol 2021;60:459-67.

64. Kewcharoen J, Techorueangwiwat C, Kanitsoraphan C, Leesutipornchai T, Akoum N, Bunch Thomas J, et al. High-power short duration and low-power long duration in atrial fibrillation ablation: A meta-analysis. J Cardiovasc Electrophysiol 2021;32:71-82.

65. Shi S, Shi J, Jia Q, Shi S, Yuan G, Hu Y. Efficacy of Physical Exercise on the Quality of Life, Exercise Ability, and Cardiopulmonary Fitness of Patients With Atrial Fibrillation: A Systematic Review and Meta-Analysis. Front Physiol 2020;11:740.

66. Atti V, Turagam MK, Garg J, Lakkireddy D. Renal sympathetic denervation improves clinical outcomes in patients undergoing catheter ablation for atrial fibrillation and history of hypertension: A meta-analysis. J Cardiovasc Electrophysiol 2019;30:702-8.

67. Vallakati A, Reddy M, Sharma A, Kanmanthareddy A, Sridhar A, Pillarisetti J, et al. Impact of gender on outcomes after atrial fibrillation ablation. Int J Cardiol 2015;187:12-6.

68. Li L, Wang Z-w, Li J, Ge X, Guo L-z, Wang Y, et al. Efficacy of catheter ablation of atrial fibrillation in patients with obstructive sleep apnoea with and without continuous positive airway pressure treatment: a meta-analysis of observational studies. Europace 2014;16:1309-14.

69. Ng CY, Liu T, Shehata M, Stevens S, Chugh SS, Wang X. Meta-analysis of obstructive sleep apnea as predictor of atrial fibrillation recurrence after catheter ablation. Am J Cardiol 2011;108:47-51.

70. Zhuang J, Lu Y, Tang K, Peng W, Xu Y. Influence of body mass index on recurrence and quality of life in atrial fibrillation patients after catheter ablation: a meta-analysis and systematic review. Clin Cardiol 2013;36:269-75.

71. Yo C-H, Lee S-H, Chang S-S, Lee MC-H, Lee C-C. Value of high-sensitivity C-reactive protein assays in predicting atrial fibrillation recurrence: a systematic review and meta-analysis. BMJ Open. 2014;4:e004418.

72. Peng H, Yang Y, Zhao Y, Xiao H. The effect of statins on the recurrence rate of atrial fibrillation after catheter ablation: A meta-analysis. Pacing Clin Electrophysiol 2018;41:1420-7.

73. Dentali F, Gianni M, Squizzato A, Ageno W, Castiglioni L, Maroni L, et al. Use of statins and recurrence of atrial fibrillation after catheter ablation or electrical cardioversion. A systematic review and meta-analysis. Thromb Haemost 2011;106:363-70.

74. Cao H, Wang X, Huang H, Ying SZ, Gu YW, Wang T, et al. Omega-3 fatty acids in the prevention of atrial fibrillation recurrences after cardioversion: a meta-analysis of randomized controlled trials. Intern Med 2012;51:2503-8.

75. Schneider MP, Hua TA, Böhm M, Wachtell K, Kjeldsen SE, Schmieder RE. Prevention of Atrial Fibrillation by Renin-Angiotensin System Inhibition: A Meta-Analysis. J Am Coll Cardiol 2010;55:2299-307.

76. Chew DS, Black-Maier E, Loring Z, Noseworthy PA, Packer DL, Exner DV, et al. Diagnosis-to-Ablation Time and Recurrence of Atrial Fibrillation Following Catheter Ablation: A Systematic Review and Meta-Analysis of Observational Studies. Circ Arrhythm Electrophysiol 2020;13:e008128.

77. Pranata R, Yonas E, Vania R. Prolonged P-wave duration in sinus rhythm pre-ablation is associated with atrial fibrillation recurrence after pulmonary vein isolation-A systematic review and meta-analysis. Ann Noninvasive Electrocardiology 2019;24:e12653.

78. Peng L, Li Z, Luo Y, Tang X, Shui X, Xie X, et al. Renin-Angiotensin System Inhibitors for the Prevention of Atrial Fibrillation Recurrence After AblatioN - A Meta-Analysis. Circ J 2020;84:1709-17.

79. Shukla A, Aizer A, Holmes D, Fowler S, Park DS, Bernstein S, et al. Effect of Obstructive Sleep Apnea Treatment on Atrial Fibrillation Recurrence: A Meta-Analysis. JACC Clin Electrophysiol 2015;1:41-51.

80. Zhao J, Liu T, Korantzopoulos P, Letsas KP, Zhang E, Yang Y, et al. Association between serum uric acid and atrial fibrillation recurrence following catheter ablation: A meta-analysis. Int J Cardiol 2016;204:103-5.

81. Pranata R, Yonas E, Chintya V, Tondas AE, Raharjo SB. Serum Galectin-3 level and recurrence of atrial fibrillation post-ablation – Systematic review and meta-analysis. Indian Pacing Electrophysiol J. 2020;20:64-9.

82. Correia ETO, Barbetta L, Silva O, Mesquita ET. Left Atrial Stiffness: A Predictor of Atrial Fibrillation Recurrence after Radiofrequency Catheter Ablation - A Systematic Review and Meta-Analysis. Arq Bras Cardiol 2019;112:501-8.

83. Njoku A, Kannabhiran M, Arora R, Reddy P, Gopinathannair R, Lakkireddy D, et al. Left atrial volume predicts atrial fibrillation recurrence after radiofrequency ablation: a meta-analysis. Europace. 2017;20:33-42.

84. Tse G, Wong CW, Gong M, Wong WT, Bazoukis G, Wong SH, et al. Predictive value of inter-atrial block for new onset or recurrent atrial fibrillation: A systematic review and meta-analysis. Int J Cardiol 2018;250:152-6.

85. Lei M, Gong M, Bazoukis G, Letsas KP, Korantzopoulos P, Li G, et al. Steroids prevent early recurrence of atrial fibrillation following catheter ablation: a systematic review and meta-analysis. Biosci Rep. 2018;38:BSR20180462.

86. Zhang N, Gong M, Tse G, Zhang Z, Meng L, Yan BP, et al. Prolonged corrected QT interval in predicting atrial fibrillation: A systematic review and meta-analysis. Pacing Clin Electrophysiol 2018;41:321-7.

87. Mclellan AJA, Kumar S, Smith C, Morton JB, Kalman JM, Kistler PM. The Role of Adenosine Following Pulmonary Vein Isolation in Patients Undergoing Catheter Ablation for Atrial Fibrillation: A Systematic Review. Journal Cardiovasc Electrophysiol 2013;24:742-51.

88. Chen W, Liu H, Ling Z, Xu Y, Fan J, Du H, et al. Efficacy of Short-Term Antiarrhythmic Drugs Use after Catheter Ablation of Atrial Fibrillation—A Systematic Review with Meta-Analyses and Trial Sequential Analyses of Randomized Controlled Trials. PLoS One 2016;11:e0156121.

89. Vitali F, Serenelli M, Airaksinen J, Pavasini R, Tomaszuk-Kazberuk A, Mlodawska E, et al. CHA2DS2-VASc score predicts atrial fibrillation recurrence after cardioversion: Systematic review and individual patient pooled meta-analysis. Clin Cardiol 2019;42:358-64.

90. Lafuente-Lafuente C, Valembois L, Bergmann JF, Belmin J. Antiarrhythmics for maintaining sinus rhythm after cardioversion of atrial fibrillation. Cochrane Database Syst Rev 2015;(3):CD005049.

91. Huang HD, Abid QU, Ravi V, Sharma P, Larsen T, Krishnan K, et al. Meta-analysis of pulmonary vein isolation ablation for atrial fibrillation conventional vs low- and zero-fluoroscopy approaches. J Cardiovasc Electrophysiol 2020;31:1403-12.

92. Aldaas OM, Lupercio F, Darden D, Mylavarapu PS, Malladi CL, Han FT, et al. Meta-analysis of the Usefulness of Catheter Ablation of Atrial Fibrillation in Patients With Heart Failure With Preserved Ejection Fraction. Am J Cardiol 2021;142:66-73.

93. Liu H, Yuan P, Zhu X, Fu L, Hong K, Hu J. Is Atrial Fibrillation Noninducibility by Burst Pacing After Catheter Ablation Associated With Reduced Clinical Recurrence?: A Systematic Review and Meta-Analysis. J Am Heart Assoc 2020;9:e015260.

94. Zhuo C, Ji F, Lin X, Jiang D, Wang L, Tian H, et al. Depression and recurrence of atrial fibrillation after catheter ablation: a meta-analysis of cohort studies. J Affect Disord 2020;271:27-32.

95. Lin KJ, Cho SI, Tiwari N, Bergman M, Kizer JR, Palma EC, et al. Impact of metabolic syndrome on the risk of atrial fibrillation recurrence after catheter ablation: systematic review and meta-analysis. J Interv Card Electrophysiol 2014;39:211-23.

96. Lee WC, Wu PJ, Fang CY, Chen HC, Chen MC. Impact of chronic kidney disease on atrial fibrillation recurrence following radiofrequency and cryoballoon ablation: A meta-analysis. Int J Clin Pract 2021;75:e14173.
